# Mental health service diversity and work disability: associations of mental health service system characteristics and mood disorder disability pensioning in Finland

**DOI:** 10.1007/s00127-023-02481-5

**Published:** 2023-04-28

**Authors:** Tino Karolaakso, Reija Autio, Petra Suontausta, Helena Leppänen, Päivi Rissanen, Turkka Näppilä, Martti T. Tuomisto, Sami Pirkola

**Affiliations:** 1 Faculty of Social Sciences (Psychology), Tampere University, Arvo Ylpön katu 34, 33520 Tampere, Finland; 2 Psychiatry, University of Helsinki and Helsinki University Hospital, Helsinki, Finland; 3 Faculty of Social Sciences (Unit of Health Sciences), Tampere University, Tampere, Finland; 4 Faculty of Medicine and Health Technology, Tampere University, Tampere, Finland; 5 Tampere University Library, Tampere University, Tampere, Finland; 6 Department of Adult Psychiatry, Tampere University Hospital, Tampere, Finland

**Keywords:** Mood disorder, Disability pension, Mental health services, Service resources, Service richness, Service diversity

## Abstract

**Purpose:**

Public mental health services (MHS) are crucial in preventing psychiatric disability pensions (DP). We studied the associations between mood disorder DP risk and the characteristics of Finnish municipalities’ MHS provision using the ESMS-R mapping tool and Finnish population registers, based on first-time granted mood disorder DPs between 2010 and 2015.

**Methods:**

The final data set included 13,783 first-time mood disorder DP recipients and 1088 mental health service units in 104 municipalities. We focused on five different MHS types: all MHS, outpatient care provision, local services without and with gatekeeping, and centralized services. Three factors for each MHS type were studied: service resources, richness, and diversity index. Negative binomial regression models were used in the analysis.

**Results:**

In all the municipalities, higher service richness and diversity regarding all MHS, outpatient care and local services with gatekeeping were associated with a lower DP risk. In urban municipalities, service richness was mainly associated with lower DP risk, and in semi-urban municipalities service diversity and resources were primarily associated with lower DP risk in outpatient care and local services with gatekeeping. In rural municipalities, DP risk indicated no association with MHS factors.

**Conclusion:**

The organization and structure of MHS play a role in psychiatric disability pensioning. MHS richness and diversity are associated with lower mood disorder DP in specific societal contexts indicating their role as quality indicators for regional MHS. The diversity of service provision should be accounted for in MHS planning to offer services matching population needs.

**Supplementary Information:**

The online version contains supplementary material available at 10.1007/s00127-023-02481-5.

## Introduction

Mental disorders are one of the most common health problems globally. In the EU, approximately 38.2% of the population suffers from a mental or neurological disorder any given year, with no substantial variation in between-country prevalence [1]. In Finland, mental and behavioral disorders are the most extensive diagnostic group from which people enter early disability retirement. In 2021, approximately 101,000 persons were on disability pension (DP) for mental disorders, which accounted for 54% of all Finnish DPs [2]. The Finnish mental disorder DPs consist mainly of depression and other mood affective disorders (F30-39) as the leading cause of early retirement (38% of all mental disorder DPs). A recent study in a similar Nordic country, Denmark, estimated that approximately 7.87 working years are lost for those with mood disorders compared to the general population [3].

Public mental health services (MHS) have a crucial role in preventing psychiatric DPs, especially for mood disorders, with timely and effective treatment. From the perspective of the health service system, a DP can be seen as a failure of the MHS to promote a person’s mental health or pre-empt and treat manifesting mental health problems. As such, the regional rate of DP recipients can be seen as one outcome indicator for the quality of services and service structure, when other regional contextual factors have been considered. The development of MHS in Finland and worldwide during recent decades has been characterized by decentralization and dehospitalization of services [4–7]. The focus of service provision has been shifted from inpatient and hospital treatment to outpatient and local community-based care since the 1980s. As a part of this shift, the provision of MHS was almost entirely transferred from the central government to municipalities in 1993, which unfortunately coincided with the severe national economic recession of the early 1990s [6, 8]. This resulted in unintended municipal divergence in the provision of MHS related to the different economic circumstances of the municipalities.

To study and implement MHS solutions successfully, it is essential to understand the health ecosystem in which they operate. Different levels and determinants of the complex system of environment and context affect regional health and mental health outcomes. Therefore consideration of the sociodemographic, -economic and -cultural contexts of the studied catchment area is essential when considering regional MHS solutions [9–14]. Previously, we have studied the Finnish district-level contextual and MHS-use related factors associated with mental disorder DP in all Finnish hospital districts [15].

To study the features of the MHS provision, a standardized description of local care delivery context comparable across different regions is required. One standardized classification system for mental health services is the European Service Mapping Schedule Revised (ESMS-R) mapping tool [9, 16]. ESMS-R provides a standardized taxonomy for describing, classifying and measuring MHS and its resources [11, 16, 17]. ESMS-R (and DESDE-LTC developed from ESMS for the similar assessment of health and social care systems) has previously been applied to compare the service systems of different countries [11, 12, 18–22] and to study the MHS within a single country or smaller regions to support evidence-informed policy making and development of the MHS [23–28]. These studies have found significant variations in care availability, capacity and gaps in care provision across geographic areas, highlighting the importance of informed MHS and policy planning for the population’s needs.

Previous research in Finland with ESMS-R data has identified that the number of different types of MHS (as different ESMS-classes, service richness) is positively associated with catchment area population size, which explains up to 84% of the service variation [23, 29]. Furthermore, studies on the characteristics of outpatient and inpatient treatment seem to refer to at least partly regionally fragmented MHS [27, 30]. A previous study has also identified that well-developed, high-quality MHS with a wider variety and higher rate of outpatient and 24 h emergency services are associated with decreased suicide rates in Finland [8]. However, the current research literature needs more information on the associations of the features of MHS structure with the risk of mood disorder DP in different municipality settings as a context. Previous studies have also focused on MHS richness, naming it service diversity but without implementing statistical diversity indices regularly used in other similar fields of study. With a more profound understanding of these associations and service diversity, regional MHS provision could be developed to prevent mental disorder-based disability more efficiently.

The aim of this study was thus to investigate the associations between mental health service system characteristics and municipal mood disorder DP risk (ICD-10 classification F30-39) in municipalities pooled to larger areas by urbanity (meso to macro level) [10, 31, 32]. We studied the effects of MHS resources, service richness and diversity, outpatient care, and local community-based and centralized services on mood disorder DP in this unique research setting, while controlling for the compositional factors of age and gender of the population. We primarily aimed to explore which MHS factors in which municipality context might be associated with mood disorder DP to produce new relevant information and research questions to promote further contextual MHS research. As a preliminary hypothesis we hypothesized that a higher rate of diversity in MHS, especially in outpatient care and community-based service, would mainly contribute to lower mood disorder DP risk, which the municipality context could moderate.

This study is part of the RETIRE – research project, which aims to study the risk factors and sequences of mental health-based disability pensioning and examine the effectiveness of service systems [15, 33–36]. The study contributes to the accumulating body of scientific knowledge needed to plan MHS to prevent work disability for mood disorders effectively.

## Methods

### Disability pension data

The study data consisted of three integrated data sets: (1) data on Finnish DP for mental disorders; (2) ESMS-R data on regional MHS from 113 Finnish municipalities within seven hospital districts; and (3) demographic information for the Finnish municipalities. The original mental disorder DP study data included all Finnish citizens granted either a temporary or permanent DP due to a mental disorder (ICD 10: F04-F69, F80-F99) for the first time between 2010 and 2015 (*N* = 50,728). The study data was collected from the registers of Statistics Finland, the Social Insurance Institution of Finland, the Finnish Centre for Pensions and the Finnish Institute for Health and Welfare (THL). During preliminary data analysis, we excluded the following subjects from the data: (1) individuals with any previous DP; (2) individuals aged under 18 or over 65; (3) individuals who had moved to a new hospital district during the last three years before receiving DP. After this exclusion process, the data included 36 879 subjects with a mental disorder DP. For a more detailed data analysis, see our previous study [33]. Lastly, for this study, we excluded the DP recipients who had been granted their DP for other than F30-39 mood disorder as their primary diagnosis and recipients living in other municipalities than those in the study area. Thus, the final data set included 13 783 first-time mood disorder DP recipients. The municipalities’ demographic characteristics were collected for 2015 from the Sotkanet Indicator Bank, an information portal provided by THL that offers essential population health and welfare data [37].

### Mental health service ESMS-R data (explanatory variables)

**Fig. 1:**
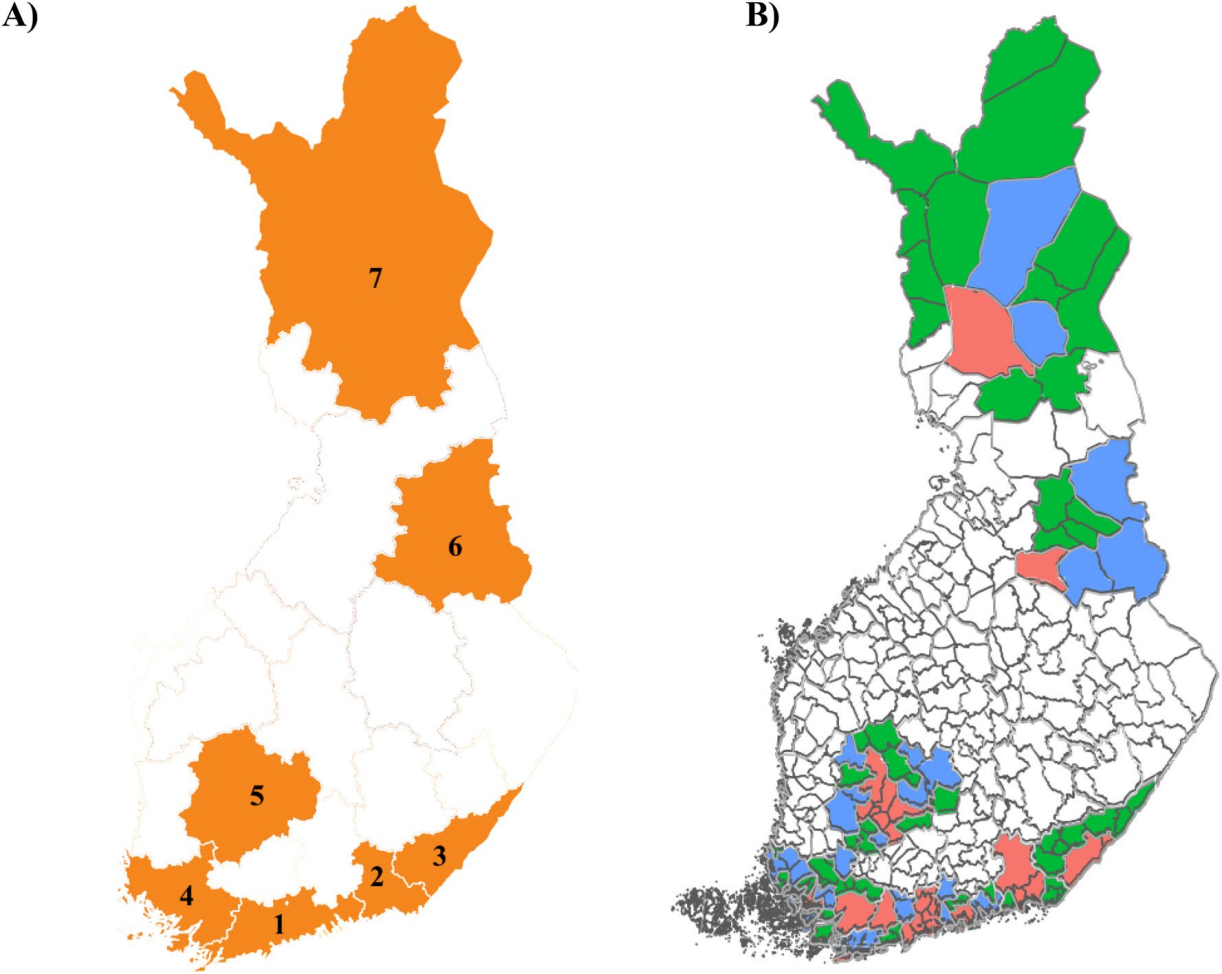
Map of the study area, comprising seven hospital districts and 113 municipalities in Finland: **A** Hospital districts 1. Helsinki and Uusimaa HUS (1 046 365 inhabitants), 2. Kymenlaakso (101 580 inhabitants), 3. South Karelia (78 248 inhabitants), 4. Southwest Finland (289 656 inhabitants), 5. Pirkanmaa (322 436 inhabitants), 6. Kainuu (43 847 inhabitants) and 7. Lapland (69 129 inhabitants). **B** Municipalities. Red: urban municipalities; Blue: semi-urban municipalities; Green: rural municipalities. Figure created with R and Inkscape

The seven hospital districts comprising the study area were Helsinki and Uusimaa (HUS), Kymenlaakso, South Karelia, Southwest Finland, Pirkanmaa, Kainuu and Lapland (Fig. 1). These hospital districts included 113 municipalities from which we excluded those with a population less than 2000 inhabitants, with the final data set having 104 municipalities. These municipalities cover approximately 60% of all Finnish citizens aged 18–64. The DP risk of these regions was reported in a previous study, which indicated that these districts are a representative sample of different Finnish regions with mostly stable DP risk [15].

The MHS of the study area were analyzed using the ESMS-R -tool [16, 17]. The ESMS-R's hierarchical taxonomy-based coding tree is described in Online Resource 1. The MHS data from HUS, Kymenlaakso, South Karelia and Southwest Finland was collected during 2012–2013 for the REFINEMENT project (Research on Financing Systems’ Effect on the Quality of Mental Health Care in Europe; for example [19, 29]). MHS in the Pirkanmaa, Lapland and Kainuu hospital districts were analyzed retrospectively for the years 2013 and 2014. These years 2012–2014 correspond with the DP data timespan of 2010–2015 in this study, as no major alterations were made to these municipal MHS during these years. This ESMS-R data is the most comprehensive available data concerning MHS system characteristics and resources in Finland.

The classification of local vs. centralized services is not initially coded in the ESMS-R taxonomy. We used a categorizing variable designed by Ala-Nikkola et al. [38] to identify local services with and without gatekeeping and centralized services, which were reclassified from the existing ESMS-R data. The Local Service variable was created using a modified Delphi panel procedure. Thus, five different MHS types from the ESMS-R -data were studied: (1) all MHS, (2) outpatient care (ESMS-R class O), (3) local services without gatekeeping, (4) local services with gatekeeping, and (5) centralized services. Outpatient services included only those services where patients were seen in an outpatient setting without the services being residential or day care services. Local services without gatekeeping included three service classes for outpatient care and four classes for day care. It also included almost all the information for care, accessibility to care and self-help and voluntary care services. Local services with gatekeeping included most of the outpatient care services, but also five classes of day care services, one information for care and one self-help and voluntary care service class. Centralized services mainly comprised day care and residential care and one type of regionally concentrated special outpatient care service class. For further details, see [38].

**Fig. 2:**
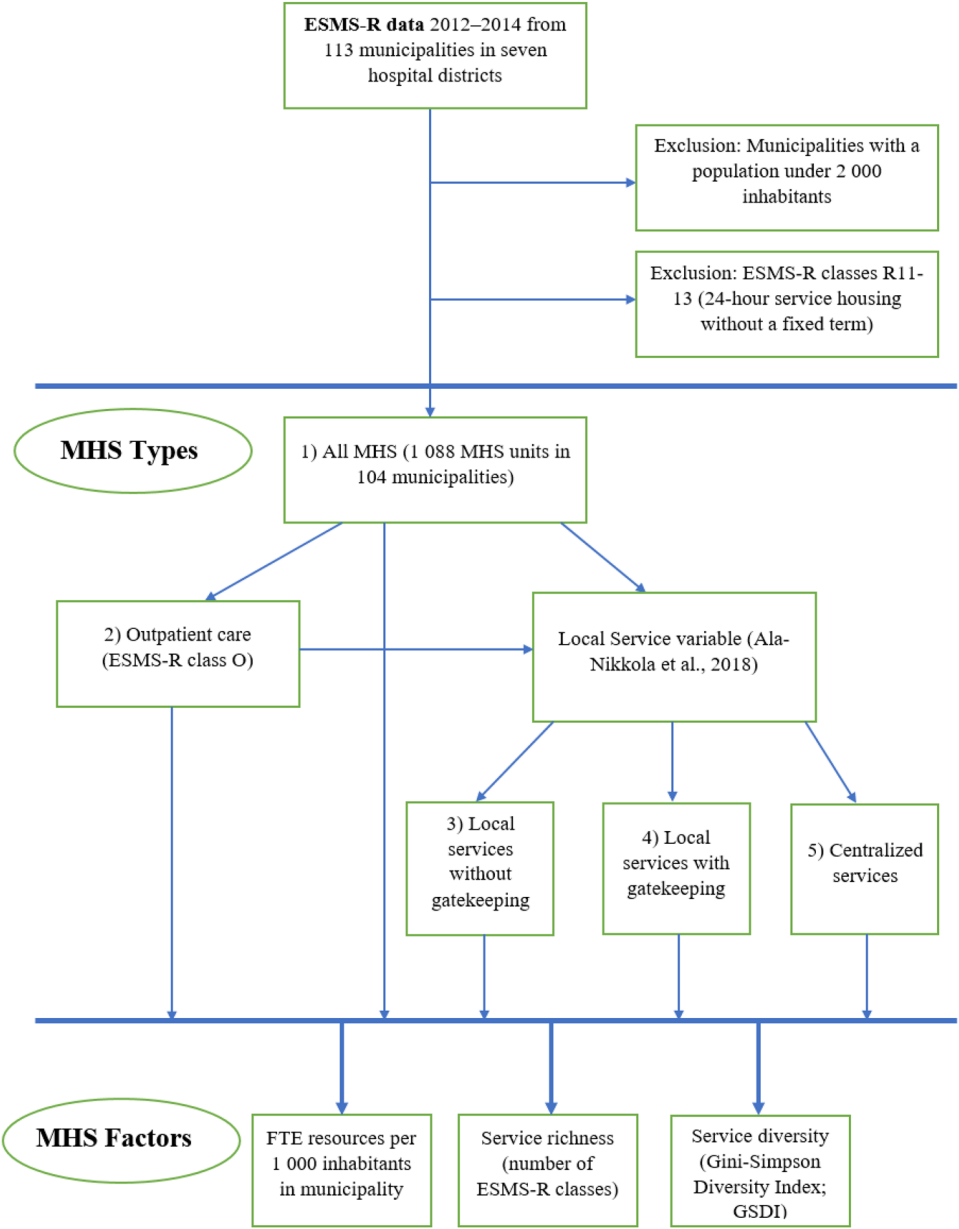
Flowchart of ESMS-R data management, the formulation of five MHS types and their three MHS factors. MHS factors were calculated for all municipalities and all MHS types. Figure created with Microsoft Office

For these five different types of MHS, three different ESMS-R -service system characteristic factors were used in the analysis: (1) service resources as the number of personnel in full-time equivalents (FTE) allocated by municipality population, per 1000 inhabitants, (2) service richness as all the different ESMS-R classes available for the municipality’s inhabitants and (3) service diversity as the Gini-Simpson Diversity Index (GSDI) calculated with service richness and the available units for the municipalities [39, 40]. The formulation of MHS types and factors from the ESMS-R data is displayed in Fig. 2.

We first calculated the sum of FTE for each municipality to evaluate the effect of the number of personnel in the MHSs. In cases where the same MHS unit provided services to inhabitants in several municipalities, the unit’s FTE was divided and allocated based on the proportion of the population in each municipality (for example, MHS X provided services with an FTE of Y to four municipalities, one of them being municipality Z. Municipality Z’s inhabitants consisted of 20% of all the persons of the area to whom MHS X provided services. Thus 20% of the Y was allocated to the municipality Z’s FTE.) Secondly, we divided the sum of FTE for each municipality by 1000 inhabitants.

Although service richness can be used as a simple way to indicate diversity, it only reflects the number of service classes reported, regardless of the number of different available service points provided for the municipalities’ inhabitants. GSDI and similar diversity indices are commonly used for example in ecological and ecosystem service research to calculate species or class diversity in a given environment or area [41]. By combining and weighing service richness with the number of available units in the ESMS-R class, the GSDI defines an index of 0 to 1 for the municipalities’ MHS diversity, with a higher GSDI signifying higher diversity. The calculation of GSDI and an example are given in Online Resource 2. The GSDI gives a more multifaceted approach to diversity of services, considering evenness between service provision rather than the mere number of ESMS-R classes commonly used in previous MHS research and named service richness in this study. To our knowledge this study is the first to examine MHS diversity using the GSDI, made possible by the ESMS-R classification of MHS classes.

Services classified as 24 h service housing without a fixed term (ESMS-R classes R11-13) were excluded from the study. These residential services presumably do not affect the disability pensioning process, as they are targeted primarily to people already on a disability pension. Furthermore, the person-years of staff information was missing from 174 services, which were excluded from the analysis regarding the service resources. However, the services with missing person-years of staff information consisted mostly of 71.3% self-help and volunteer care services (ESMS-R class S), with only 6.9% outpatient care (O) services. The final mental health service data set included 1 088 MHS units in 104 municipalities.

### Statistical analysis

The municipalities were divided into three groups based on the 2015 classification by Statistics Finland for describing the degree of urbanization of their residence: urban, semi-urban and rural. Urban municipalities included those in which at least 90% of the population lived in urban settlements or where the population of the largest urban settlement was at least 15,000. In semi-urban municipalities, at least 60% but less than 90% of the population lived in urban settlements, and the population of the largest urban settlement was between 4000 and 15,000 inhabitants. In rural municipalities, less than 60% lived in urban settlements, and the population of the largest settlement was less than 15,000 inhabitants, or between 60 and 90% of the population lived in urban settlements, and the largest settlement was less than 4000 inhabitants.

Means and standard deviations were calculated for the MHS factors to characterize the data. One-way ANOVA test was used to determine whether the municipality groups had statistically significant differences concerning the MHS factors. The associations of MHS factors with mood disorder DP were analyzed using negative binomial regression models. The regression analyses were performed by applying robust standard errors, using the Finnish population data within each municipality as an exposure and with adjustment based on the compositional factors gender and age. The information on the municipality population aged 18 to 65 and their age and gender distribution in 2015 was acquired from Statistics Finland (*N* = 1,950,205) for the 104 municipalities.

Incidence rate ratios (IRR) and 95% confidence intervals (95% CI) were calculated for the models. Because of varying multicollinearity between the different MHS types and municipality groups, the analysis for each MHS factor was modeled separately. The correlation between the municipality groups MHS factors and demographic characteristics are shown in Online Resource 3. In all statistical analyses, *P* values < 0.05 were considered statistically significant. GSDI values were calculated for the MHS factors with R version 4.0.1 [42], RStudio [43] and the R-package diverse [44]. The statistical analyses were performed with SPSS version 28.0.

## Results

### Study area and MHS factor characteristics

Characteristics of the study’s catchment area and municipality groups on the macro-level are reported in Table 1. Urban municipalities comprised 29% of all the municipalities, but 79% of all DP recipients and 81% of all the catchment area population resided in them. Urban municipalities also had a lower ratio of mood disorder DP, mental health index and dependency ratio in the population, as well as a higher rate of higher education qualifications and population density. The characteristics of the semi-urban and rural municipalities were primarily similar, although semi-urban municipalities had lower unemployment rates. Rural municipalities also had the lowest rates of population density and higher education qualifications, but also the lowest rate of those not in education or training at age 17–24.

**Table 1:** Demographic characteristics of the municipalities in the study (2015)

	All municipalities	Urban municipalities	Semi-urban municipalities	Rural municipalities
Municipalities included in study	104	30	26	48
First time mood disorder F30-39 DP receivers 2010–2015	13 783	10 943	1 872	968
Total population aged 18 to 65, end of 2015	1 951 261	1 584 015	240 458	126 788
Ratio of mood disorder DP, % of population aged 18 to 65	0.71%	0.69%	0.78%	0.76%
Mental health index, not age-standardized*	98.8 (39.7–184)	94.5 (52.7–126.9)	100.4 (52.8–136.9)	100.7 (39.7–184)
Unemployment rate, as % of total population*	12.8% (6.8–22.9%)	13.1% (7.6–19.9%)	12.1% (6.8–20.1%)	13.0% (7–22.9%)
Household-dwelling-units with one person, as % of all household/dwelling-units*	38.9% (22.2–51.2%)	39.8% (29.9–51.1%)	38.5% (23.9–45.8%)	38.6% (22.2–51.2%)
Population density, population/km^2^*	47.4 (0.5–2936.6)	407.4 (8.2–2936.6)	31.2 (0.8–115.8)	11 (0.5–48.1)
Demographic dependency ratio, as the number of people aged under 15 and over 64 per hundred working-age people aged 15–64*	67.3 (44–102.8)	58.9 (44–72.3)	67.6 (57–79.9)	72.4 (55.2–102.8)
Higher education qualifications, as % of total population aged 20 and over*	25.0% (13.8–57.1%)	31.7% (21–57.1%)	24.8% (16–35%)	21% (13.8–34.4%)
Not in education or training aged 17–24, as % of total population of same age*	8.6% (3.5–16%)	8.9% (5.5–15%)	9% (5.4–14.3%)	8.1% (3.5–16%)

*Mean (and range) for the municipalities

Means and standard deviations are reported for the MHS types and their factors in Table 2 and Online Resource 4. In all MHS types the municipality groups were statistically significant concerning service richness and diversity, but did not differ regarding FTE resources per 1000 inhabitants. The mean number of MHS FTE in all municipalities was 3.13 per 1000 inhabitants (SD 1.28). The mean for service richness of all MHS was 15.67 distinct ESMS-R classes offering services to a single municipality’s residents (SD 6.09). The mean value of GSDI for all MHS diversity was 0.88 (SD 0.04) between all municipalities. Concerning the municipality groups and MHS types, service richness and diversity were highest in urban municipalities, while being lower in semi-urban and typically lowest in rural municipalities (Table 2). Only in local services with gatekeeping did rural municipalities have a higher GSDI (0.69; SD 0.11) than semi-urban (0.66; SD 0.18).

**Table 2:** Characteristics of the municipality-level mental health service ESMS-R factors in Finland as means (with standard deviation)

	All municipalities	Urban municipalities	Semi-urban municipalities	Rural municipalities	Statistical significance^d^
*All mental health services (MHS)*					
FTE resources per 1000 inhabitants^a^	3.13 (1.28)	3.12 (0.91)	2.96 (1.33)	3.23 (1.43)	*p* = 0.688
Service richness^b^	15.67 (6.09)	21.4 (6.38)	14.81 (4.28)	12.56 (3.82)	*p* < 0.001
Service diversity^c^	0.88 (0.04)	0.91 (0.02)	0.88 (0.05)	0.86 (0.04)	*p* < 0.001
*Outpatient care (ESMS-R code O)*					
FTE resources per 1000 inhabitants^a^	1.30 (0.58)	1.27 (0.34)	1.27 (0.57)	1.34 (0.69)	*p* = 0.843
Service richness^b^	5.57 (1.90)	7.00 (2.16)	5.35 (1.42)	4.79 (1.36)	*p* < 0.001
Service diversity^c^	0.70 (0.10)	0.74 (0.07)	0.72 (0.15)	0.67 (0.07)	*p* = 0.009
*Local services without gatekeeping*					
FTE resources per 1000 inhabitants^a^	0.63 (0.54)	0.61 (0.52)	0.68 (0.48)	0.61 (0.57)	*p* = 0.871
Service richness^b^	3.80 (2.08)	5.57 (2.24)	3.81 (1.36)	2.69 (1.44)	*p* < 0.001
Service diversity^c^	0.54 (0.26)	0.71 (0.09)	0.60 (0.21)	0.40 (0.28)	*p* < 0.001
*Local services with gatekeeping*					
FTE resources per 1000 inhabitants^a^	0.90 (0.67)	0.89 (0.46)	0.78 (0.67)	0.97 (0.76)	*p* = 0.515
Service richness^b^	5.15 (2.27)	7.03 (2.82)	4.58 (1.67)	4.29 (1.24)	*p* < 0.001
Service diversity^c^	0.71 (0.13)	0.77 (0.07)	0.66 (0.18)	0.69 (0.11)	*p* = 0.002
*Centralized services*					
FTE resources per 1000 inhabitants^a^	1.60 (0.79)	1.62 (0.52)	1.50 (0.82)	1.65 (0.91)	*p* = 0.757
Service richness^b^	6.72 (2.78)	8.80 (2.87)	6.42 (2.31)	5.58 (2.18)	*p* < 0.001
Service diversity^c^	0.74 (0.13)	0.80 (0.06)	0.73 (0.16)	0.72 (0.14)	*p* = 0.028

^a^Resources as the number of personnel in full-time equivalents (FTE) allocated by municipality population, per 1000 inhabitants

^b^Richness as all the different ESMS-R -classes available for the municipality’s inhabitants

^c^Diversity as the Gini-Simpson Diversity Index (GSDI), calculated with service richness and the available units for the municipalities

^d^Statistical significances to detect whether the mean values of the urban, semi-urban and rural municipalities were different were computed with the one-way ANOVA test

### Mood disorder disability pensioning and mental health services

Noticeable differences between MHS factors and mood disorder DP associations were observed (Table 3). The relationship between MHS factors and DP appears to be associated with the degree of urbanicity and the context of the municipalities. When all municipalities were studied together, a higher service richness and diversity in all MHS (richness IRR 0.995; 95% CI 0.991–0.998, and GSDI IRR 0.396; 95% CI 0.185–0.850), outpatient care (richness IRR 0.978; 95% CI 0.996–0.990, and GSDI IRR 0.687; 95% CI 0.511–0.924) and local services with gatekeeping (richness IRR 0.980; 95% CI 0.970–0.990, and GSDI IRR 0.641; 95% CI 0.500–0.821) were associated with lower DP risk.

**Table 3:** Associations of mental health service ESMS-R factors with mood disorder (F30-39) DP in Finland by incidence rate ratio (IRR) and 95% confidence interval (95% CI)

	All municipalities (*N* = 104)	Urban municipalities (*n* = 30)	Semi-urban municipalities (*n* = 26)	Rural municipalities (*n* = 48)
	IRR (95% CI)	IRR (95% CI)	IRR (95% CI)	IRR (95% CI)
*All mental health services (MHS)*				
FTE resources per 1000 inhabitants^a^	0.997 (0.965–1.030)	1.026 (0.970–1.085)	0.941 (0.897–0.988)*	1.046 (0.993–1.102)
Service richness^b^	0.995 (0.991–0.998)**	0.993 (0.988–0.998)**	0.998 (0.981–1.014)	0.996 (0.978–1.013)
Service diversity^c^	0.396 (0.185–0.850)*	0.211 (0.035–1.284)	0.373 (0.121–1.155)	0.555 (0.070–4.388)
*Outpatient Care (ESMS-R code O)*				
FTE resources per 1000 inhabitants^a^	0.991 (0.918–1.070)	1.091 (0.962–1.237)	0.818 (0.726–0.922)**	1.109 (0.984–1.251)
Service richness^b^	0.978 (0.966–0.990)**	0.976 (0.962–0.990)**	0.961 (0.918–1.006)	0.985 (0.933–1.040)
Service diversity^c^	0.687 (0.511–0.924)*	0.679 (0.423–1.092)	0.644 (0.442–0.940)*	1.053 (0.408–2.716)
*Local services without gatekeeping*				
FTE resources per 1000 inhabitants^a^	1.021 (0.953–1.094)	0.988 (0.901–1.084)	0.948 (0.824–1.091)	1.124 (0.995–1.270)
Service richness^b^	0.990 (0.979–1.001)	0.985 (0.970–1.000)*	1.019 (0.967–1.074)	1.007 (0.958–1.058)
Service diversity^c^	0.905 (0.778–1.054)	0.428 (0.258–0.711)**	0.925 (0.660–1.298)	1.083 (0.845–1.387)
*Local services with gatekeeping*				
FTE resources per 1000 inhabitants^a^	0.974 (0.914–1.038)	1.050 (0.946–1.165)	0.881 (0.792–0.980)*	0.997 (0.890–1.116)
Service richness^b^	0.980 (0.970–0.990)**	0.982 (0.971–0.994)**	0.943 (0.913–0.975)**	0.943 (0.887–1.001)
Service diversity^c^	0.641 (0.500–0.821) **	0.865 (0.489–1.531)	0.544 (0.398–0.743)**	0.673 (0.327–1.383)
*Centralized services*				
FTE resources per 1000 inhabitants^a^	0.997 (0.947–1.050)	1.038 (0.945–1.140)	0.925 (0.859–0.996)*	1.058 (0.976–1.147)
Service richness^b^	0.994 (0.985–1.003)	0.986 (0.974–0.998)*	1.021 (0.989–1.054)	1.008 (0.977–1.040)
Service diversity^c^	0.821 (0.613–1.099)	1.079 (0.548–2.126)	0.756 (0.500–1.145)	0.898 (0.546–1.477)

Negative binomial regression model adjusted based on the compositional factors gender and age

^a^Resources as the number of personnel in full-time equivalents (FTE) allocated by municipality population, per 1000 inhabitants

^b^Richness as all the different ESMS-R -classes available for the municipality’s inhabitants

^c^Diversity as the Gini-Simpson Diversity Index (GSDI), calculated with service richness and the available units for the municipalities

*Statistical significance at the 0.05 level

**Statistical significance at the 0.01 level

In urban municipalities service richness was associated with lower DP in all five studied MHS types (all MHS IRR 0.993; 95% CI 0.988–0.998, outpatient care IRR 0.976; 95% CI 0.962–0.990, local services without gatekeeping IRR 0.985; 95% CI 0.970–1.000, with gatekeeping IRR 0.982; 95% CI 0.971–0.994, and centralized services IRR 0.986; 95% CI 0.974–0.998), as well as with service diversity in local services without gatekeeping (IRR 0.428; 95% CI 0.258–0.711).

Uniquely in semi-urban municipalities, a higher FTE per 1000 inhabitants indicated a lower DP risk in all MHS (IRR 0.941; 95% CI 0.897–0.988), outpatient care (IRR 0.818; 95% CI 0.726–0.922), local services with gatekeeping (IRR 0.881; 95% CI 0.792–0.980) and centralized services (IRR 0.925; 95% CI 0.859–0.996), but not in local services without gatekeeping. Furthermore, in outpatient care we found a lower risk of DP associated with higher service diversity (IRR 0.644; 95% CI 0.442–0.940), and in local services with gatekeeping a lower DP risk with higher service richness (IRR 0.943; 95% CI 0.913–0.975) and diversity (IRR 0.544; 95% CI 0.398–0.743). Thus, all studied MHS factors showed an association with lower DP risk in local services with gatekeeping, but not in local services without gatekeeping. Interestingly, we found no associations between rural municipalities' DP risk and MHS factors.

## Discussion

In this comprehensive population-level study, we found significant associations between the resourcing, service richness and diversity of MHS and the level of mood disorder DP. Our associations illustrate differences in distinct municipality contexts. These findings suggest that the organization and structure of available MHS are associated with the incidence of psychiatric disability pensioning.

A novel approach to using the GSDI enabled us to identify an association of higher service richness and diversity with lower DP risk, especially in all MHS, outpatient care and local services with gatekeeping. Higher service richness and diversity in these MHS types may be indicators of a well-developed, high-quality service system with higher effectiveness in the pre-emption of disability due to mood disorders. Higher diversity in MHS could also result in services responding more broadly to different population demands and having fewer gaps in service provision for the needs of the population [8]. This was evident in the semi-urban municipality context and when examining all municipalities together, and might also be partly conveyed in urban municipalities by service richness, but interestingly not by service diversity. Lower service richness and diversity might result in critical systemic gaps in the provision of MHS and care pathways. Prior studies have identified some of these gaps using ESMS-R or DESDE-LTC taxonomies [19, 21, 22, 26, 29].

In a high-income Nordic country such as Finland, there are clear differences between the urban, semi-urban and rural contexts of MHS provision. On average, the diversity of MHS is higher, and mood disorder DP risk is lower in larger municipalities, which may reflect the historical and economic background in the provision and organization of MHS by Finnish municipalities. The effects of MHS factors appear to be most clearly associated with mood disorder DP in a semi-urban context. This might indicate that other contextual factors do not affect mood disorder DP differences to the extent that changes in regional service provision would have the potential to be essential or main contributors. In urban and rural municipalities, other sociodemographic and contextual economic factors might significantly affect mood disorder DP rates and populational needs for the MHS. The urban municipalities in our study had, on average, a lower dependency ratio in the population and a higher rate of higher education qualifications. It is also important to note that in urban municipalities the MHS includes more service units and system components which need to be interconnected. MHS comprise complex dynamic systems, and the (un)successful organization of this complexity for effective patient care pathways might be one confounding factor in the provision of large urban area MHS systems [10, 13, 14].

Interestingly, rural municipalities did not have significant associations with MHS factors. Possible related contextual factors might be that rural municipalities had the lowest rates of population density (which associates with longer distances to the physical location of service provision [15]) and of higher education qualifications but also of those not in education or training at age 17–24. In addition, a higher average age of population, higher unemployment rate, and emphasis on blue-collar occupations was often associated with a rural context, which might have confounding effects on regional mood disorder DP risk.

### Strengths, limitations and future research

The strengths of this study include the use of comprehensive national-level data registers. Finnish population registers have high coverage and quality, allowing detailed and extensive epidemiologic research for MHS associations with mood disorder DP in this study [45, 46]. To our knowledge, there has not previously been a comprehensive study of the relationship between MHS types and factors, and mood disorder DP in different municipality contexts. The ESMS-R mapping tool was used for clear hierarchical taxonomy-based coding of MHS. This study also included an examination of the MHS context of service provision. Both are essential in researching complex MHS systems [9, 10, 16].

Our study setting includes some important limitations. One major limitation in this study was that because of the varying multicollinearity between the different MHS types and municipality groups, not all the MHS factors could be entered and adjusted in the same model. The correlation between the municipality groups MHS factors and demographic characteristics are provided in Online Resource 3. Secondly, the MHS units could be of different sizes, which does not affect the FTE but could affect GSDIs, which were calculated with the available units in the municipality and therefore reflected the number of components in the complex MHS system rather than the components’ size. Thirdly, some MHS units provided services to several municipalities, which could involve regional dynamics that were not comprehensively considered in this study. In these MHSs, the FTE resources were allocated to municipalities on the basis of their relative share of inhabitants. This factor is based on the assumption that all the municipality’s inhabitants used the MHS available to them in equal amounts. However, this might not be the case, although this was the best available estimate in this study. Fourthly, the MHS data does not include information about the co-operation of the services or pathways of care between them, or on whether the psychosocial treatment provided was grounded in evidence-based psychosocial treatment models and a specific philosophy/culture of psychosocial treatment provision. In future research, the treatment contents and cultures should be integrated to ESMS-R classification research, which could yield a more complex but truthful picture of MHS ecosystems and functioning concerning mood disorder treatment and DP prevention.

It is important to note that the ESMS-R classification tool is not all-encompassing, and there may have been subtle features of the classified MHS units that are not included in the analysis. The ESMS-R data in this study only includes public services, and it excludes Finnish occupational health care, private services or rehabilitative psychotherapy imbursed by the Social Insurance Institution of Finland. However, previous studies have indicated a lower mental disorder DP rate in occupational health care users compared to population statistics [47], and a higher rate of sick leaves for mental disorders in public service users compared to occupational health or private service users [48]. These findings indicate that public MHS have a crucial populational role in treating and pre-empting mental disorders and disability for most of the population.

## Conclusions

Our findings of significant associations between MHS factors, especially service richness and diversity, with mood disorder DP in Finnish municipalities highlight the importance of organizational factors for the effectiveness of services. Higher service richness and diversity in all MHS, outpatient care and community-based services may be indicators of a well-developed high-quality service system with a higher effectiveness in pre-emption of mood disorder DP. Higher diversity of MHS could support a broader response to different populational needs and leave fewer gaps in treatment provision. There are also differences between the urban, semi-urban and rural contexts of the MHS provision, which might be connected to other confounding contextual factors, especially in many urban or rural environments.

The Finnish Mental Health Strategy 2020–2030 promotes broad-based MHS that meet people’s needs, highlighting the requirement that the services should be of high accessibility, effectivity, quality, availability, flexibility, and compatibility and that they should support continuity [49]. There are already several such programs and initiatives in Finland, aiming to elevate the contents and care pathways of regional MHS [50, 51]. The diversity of service provision should be accounted for in MHS planning by experts and stakeholders to offer services matching population needs.

### Supplementary Information

Below is the link to the electronic supplementary material.

Supplementary file1 (PDF 161 KB)

Supplementary file2 (PDF 115 KB)

Supplementary file3 (PDF 632 KB)

Supplementary file4 (PDF 241 KB)

## Data Availability

The used DP recipient and population datasets are available from Statistics Finland, the Social Insurance Institution of Finland, the Finnish Centre for Pensions and the Finnish Institute for Health and Welfare. Restrictions apply to the availability of these data, which were used under license for the current study, and so are not publicly available. ESMS-R data is available from the Finnish Institute for Health and Welfare. The municipality-level factors are publicly available from the Sotkanet Indicator Bank, an information portal provided by THL, and the datasets used and analyzed during the current study are available from the corresponding author on reasonable request.
