# Supplementary material for: Mental health service diversity and work disability: associations of mental health service system characteristics and mood disorder disability pensioning in Finland
Source: Soc Psychiatry Psychiatr Epidemiol. 2023 Apr 28;59(4):631–42. doi: 10.1007/s00127-023-02481-5 (PMC10960744; doi:10.1007/s00127-023-02481-5)
Supplement: Supplementary file 1 — Supplementary file1 (PDF 161 KB) [file 127_2023_2481_MOESM1_ESM.pdf]

Online Resource 1. ESMS-R classification mapping tree. Includes the Local Service variable: A = local services without gatekeeping, B = local services with gatekeeping, C = centralized services.

Author information: Mental health service diversity and work disability: associations of mental health service system characteristics and mood disorder disability pensioning in Finland. (2023). Social Psychiatry and Psychiatric Epidemiology. Tino Karolaakso [Faculty of Social Sciences (Psychology), Tampere University, Arvo Ylpön katu, 34, 33520 Tampere, Finland; tino.karolaakso@tuni.fi], Reija Autio, Petra Suontausta. Helena Leppänen, Päivi Rissanen, Turkka Näppilä, Martti T. Tuomisto, Sami Pirkola.

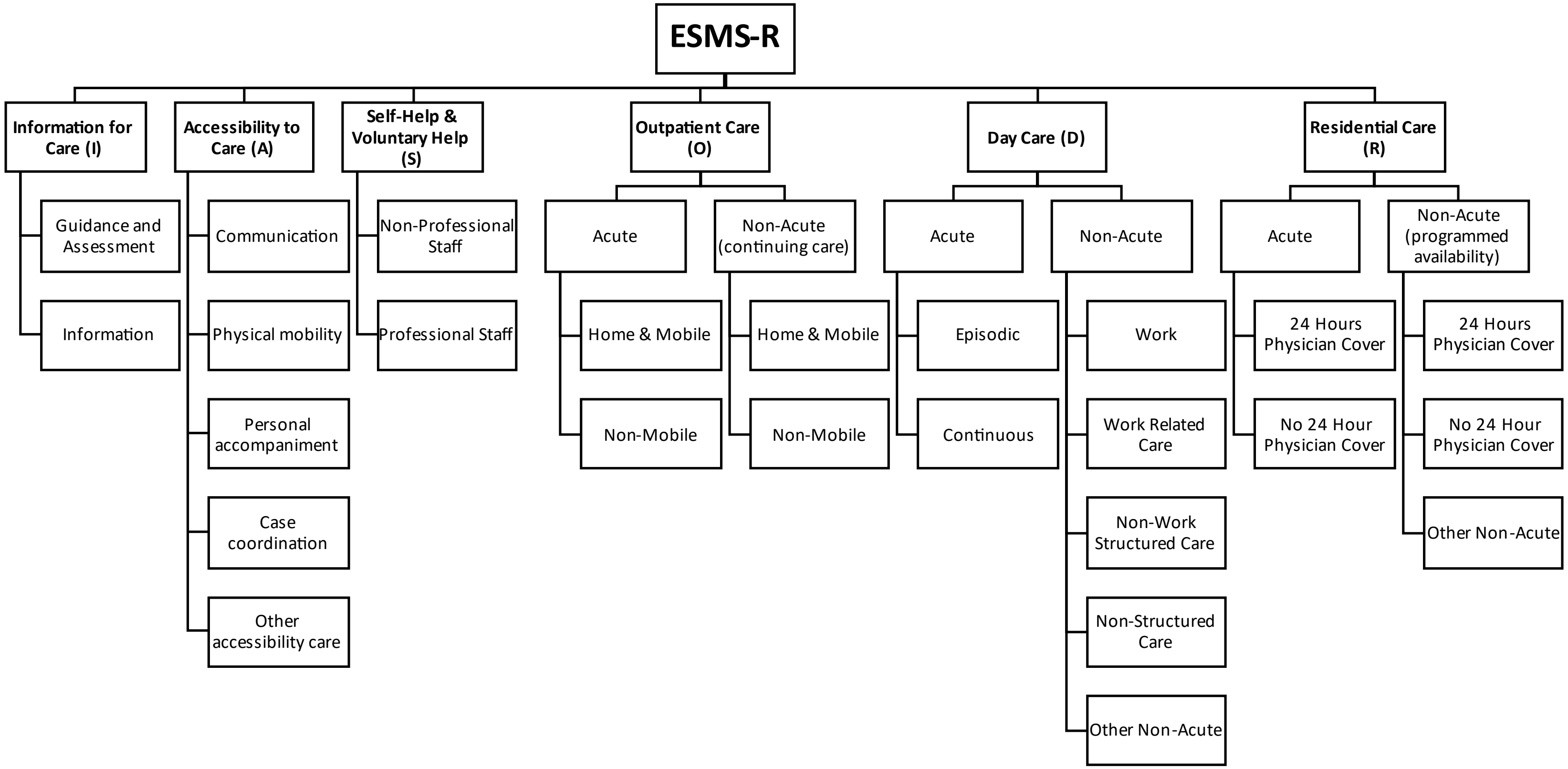

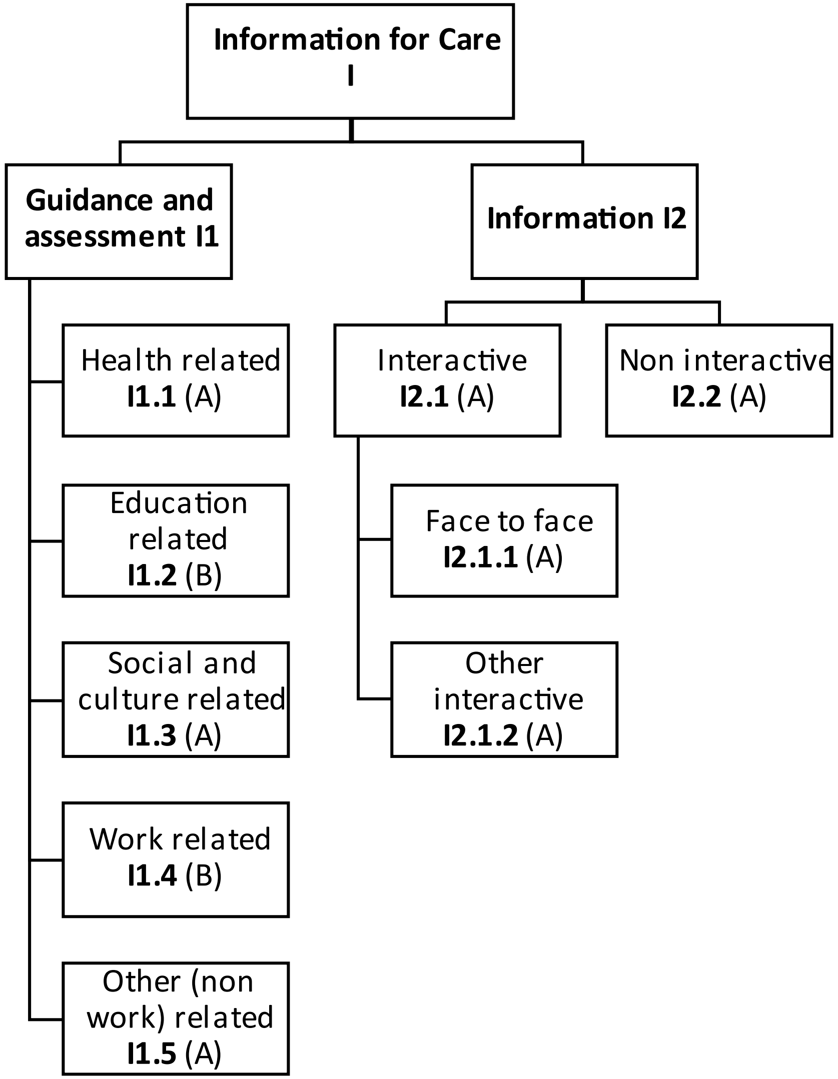
Information for Care
I
Guidance and assessment I1
Information I2
Health related
I1.1 (A)
Education related
I1.2 (B)
Social and culture related
I1.3 (A)
Work related
I1.4 (B)
Other (non work) related
I1.5 (A)
Interactive
I2.1 (A)
Non interactive
I2.2 (A)
Face to face
I2.1.1 (A)
Other interactive
I2.1.2 (A)

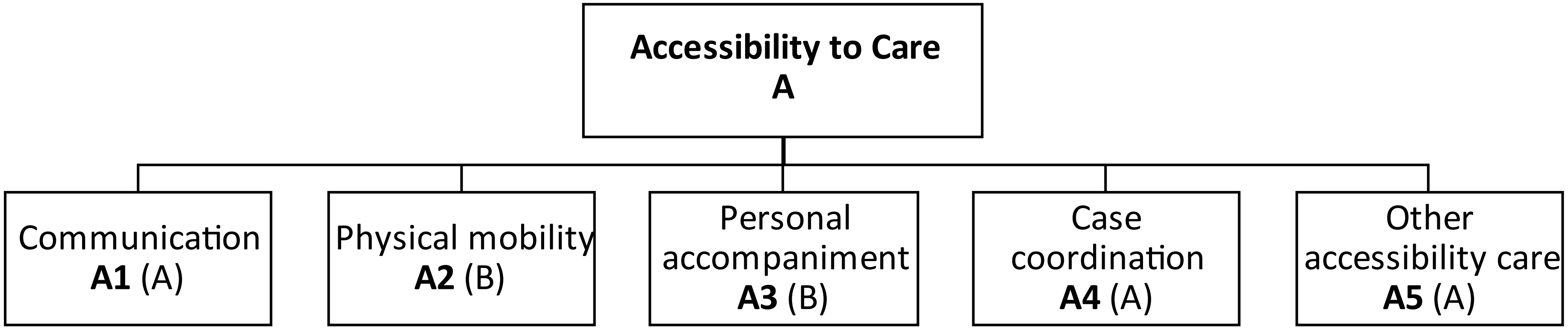
Accessibility to Care
A
Communication
A1 (A)
Physical mobility
A2 (B)
Personal accompaniment
A3 (B)
Case coordination
A4 (A)
Other accessibility care
A5 (A)

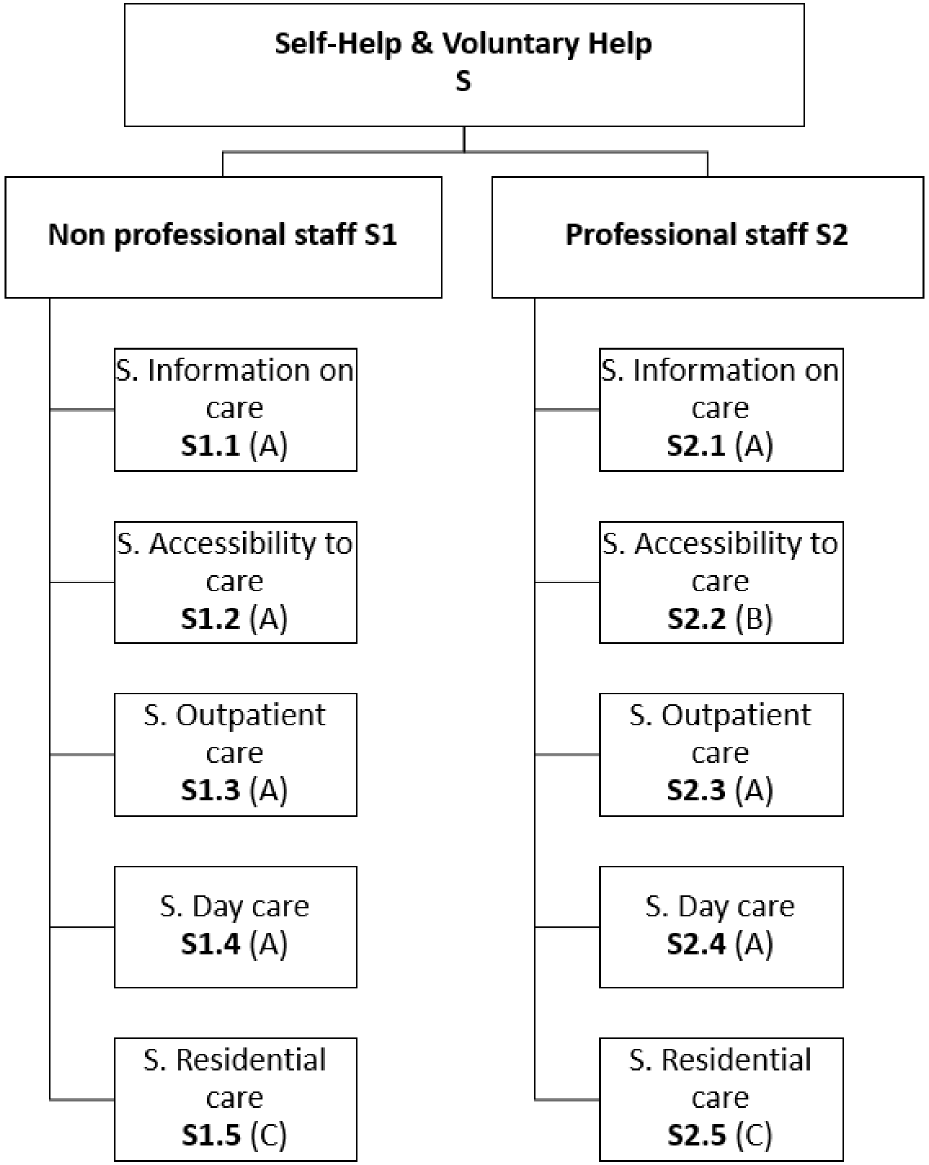
Self-Help & Voluntary Help
S
Non professional staff S1
Professional staff S2
S. Information on care
S1.1 (A)
S. Accessibility to care
S1.2 (A)
S. Outpatient care
S1.3 (A)
S. Day care
S1.4 (A)
S. Residential care
S1.5 (C)
S. Information on care
S2.1 (A)
S. Accessibility to care
S2.2 (B)
S. Outpatient care
S2.3 (A)
S. Day care
S2.4 (A)
S. Residential care
S2.5 (C)

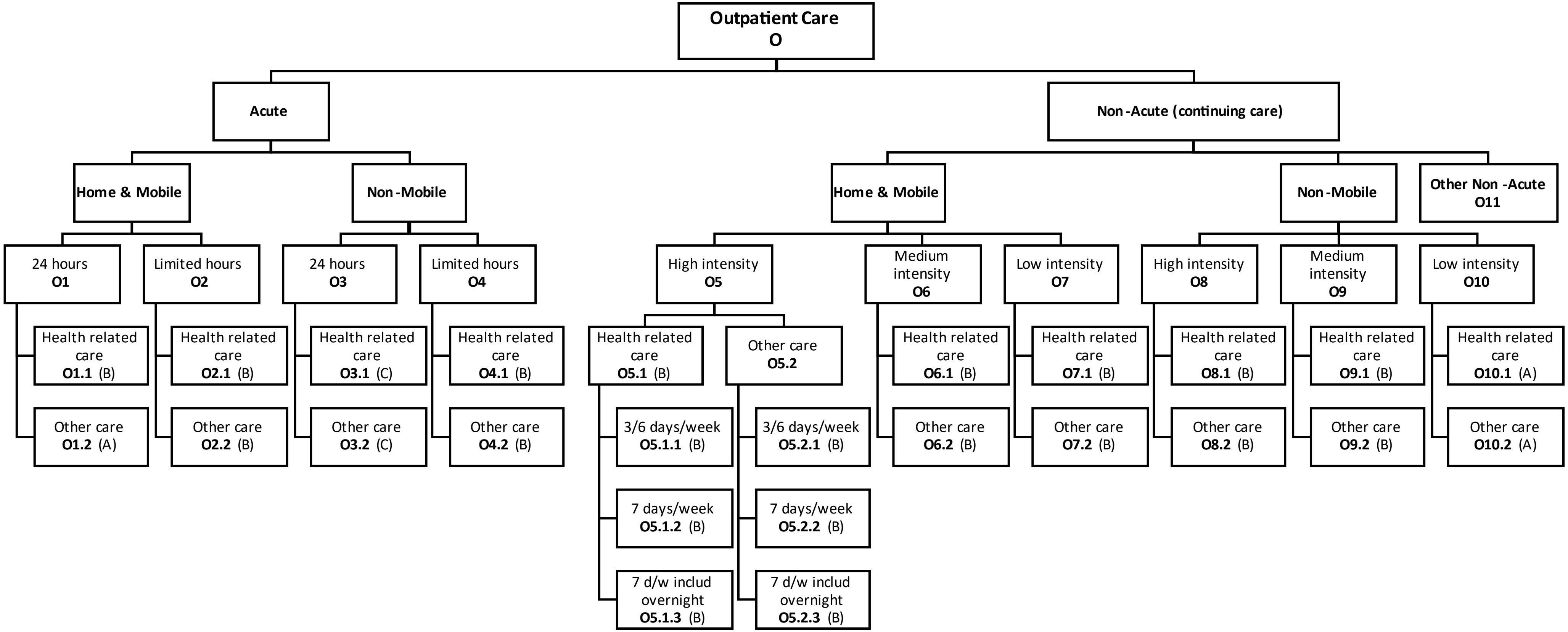

Outpatient Care O
Acute
Non-Acute (continuing care)
Home & Mobile
Non-Mobile
Home & Mobile
Non-Mobile
Other Non-Acute O11
24 hours O1
Limited hours O2
24 hours O3
Limited hours O4
High intensity O5
Medium intensity O6
Low intensity O7
High intensity O8
Medium intensity O9
Low intensity O10
Health related care O1.1 (B)
Other care O1.2 (A)
Health related care O2.1 (B)
Other care O2.2 (B)
Health related care O3.1 (C)
Other care O3.2 (C)
Health related care O4.1 (B)
Other care O4.2 (B)
Health related care O5.1 (B)
Other care O5.2
3/6 days/week O5.1.1 (B)
7 days/week O5.1.2 (B)
7 d/w includ overnight O5.1.3 (B)
3/6 days/week O5.2.1 (B)
7 days/week O5.2.2 (B)
7 d/w includ overnight O5.2.3 (B)
Health related care O6.1 (B)
Other care O6.2 (B)
Health related care O7.1 (B)
Other care O7.2 (B)
Health related care O8.1 (B)
Other care O8.2 (B)
Health related care O9.1 (B)
Other care O9.2 (B)
Health related care O10.1 (A)
Other care O10.2 (A)

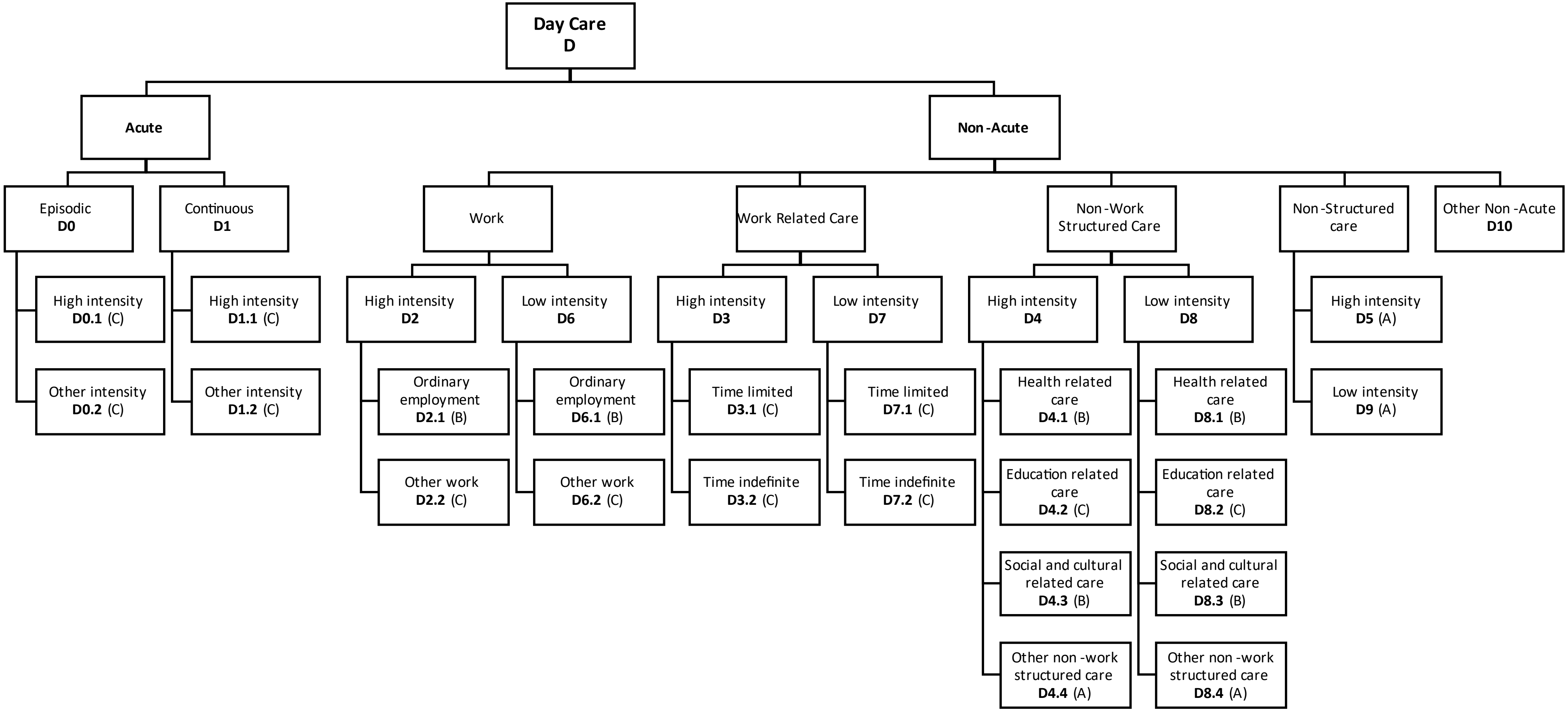
Day Care
D
Acute
Non -Acute
Episodic
D0
Continuous
D1
Work
Work Related Care
Non -Work
Structured Care
Non -Structured
care
Other Non -Acute
D10
High intensity
D0.1 (C)
Other intensity
D0.2 (C)
High intensity
D1.1 (C)
Other intensity
D1.2 (C)
High intensity
D2
Low intensity
D6
High intensity
D3
Low intensity
D7
High intensity
D4
Low intensity
D8
High intensity
D5 (A)
Low intensity
D9 (A)
Ordinary
employment
D2.1 (B)
Other work
D2.2 (C)
Ordinary
employment
D6.1 (B)
Other work
D6.2 (C)
Time limited
D3.1 (C)
Time indefinite
D3.2 (C)
Time limited
D7.1 (C)
Time indefinite
D7.2 (C)
Health related
care
D4.1 (B)
Education related
care
D4.2 (C)
Social and cultural
related care
D4.3 (B)
Other non -work
structured care
D4.4 (A)
Health related
care
D8.1 (B)
Education related
care
D8.2 (C)
Social and cultural
related care
D8.3 (B)
Other non -work
structured care
D8.4 (A)

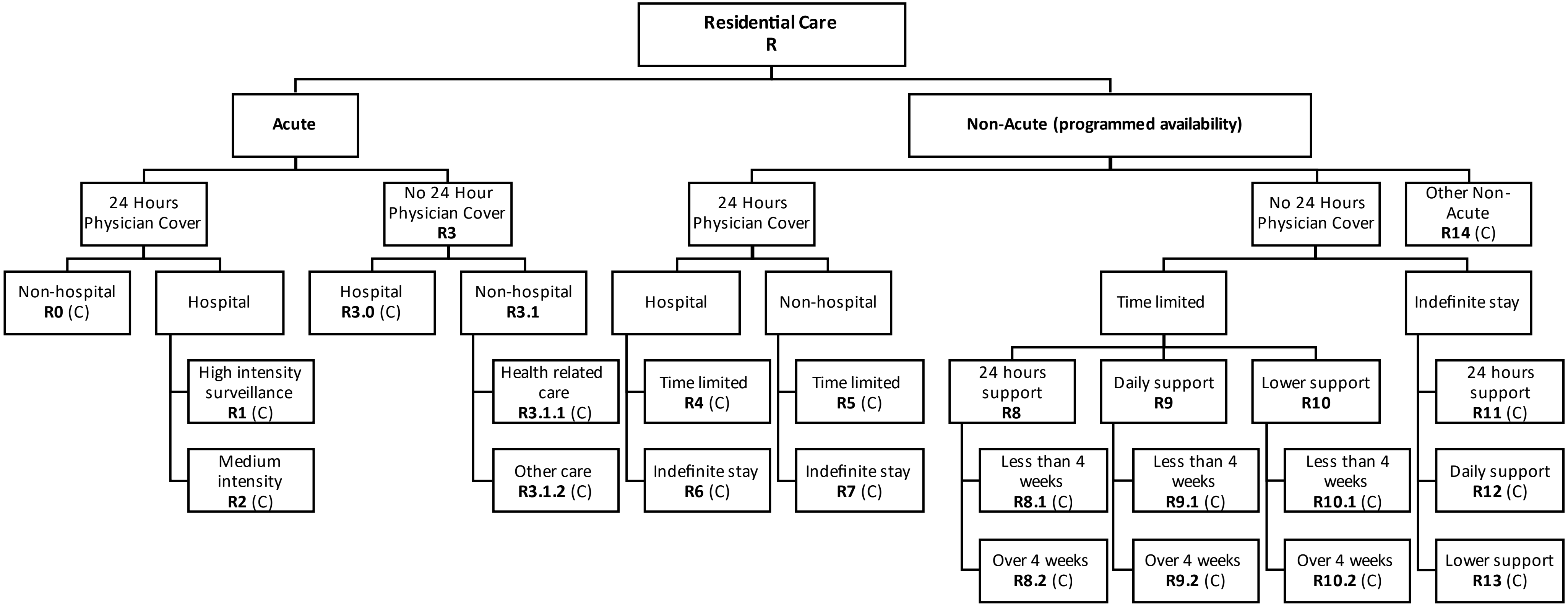

Residential Care
R
Acute
Non-Acute (programmed availability)
24 Hours Physician Cover
No 24 Hour Physician Cover R3
24 Hours Physician Cover
No 24 Hours Physician Cover
Other Non-Acute R14 (C)
Non-hospital R0 (C)
Hospital
Hospital R3.0 (C)
Non-hospital R3.1
Hospital
Non-hospital
Time limited
Indefinite stay
High intensity surveillance R1 (C)
Medium intensity R2 (C)
Health related care R3.1.1 (C)
Other care R3.1.2 (C)
Time limited R4 (C)
Indefinite stay R6 (C)
Time limited R5 (C)
Indefinite stay R7 (C)
24 hours support R8
Daily support R9
Lower support R10
Less than 4 weeks R8.1 (C)
Over 4 weeks R8.2 (C)
Less than 4 weeks R9.1 (C)
Over 4 weeks R9.2 (C)
Less than 4 weeks R10.1 (C)
Over 4 weeks R10.2 (C)
24 hours support R11 (C)
Daily support R12 (C)
Lower support R13 (C)
