# Supplementary material for: Mental health service diversity and work disability: associations of mental health service system characteristics and mood disorder disability pensioning in Finland
Source: Soc Psychiatry Psychiatr Epidemiol. 2023 Apr 28;59(4):631–42. doi: 10.1007/s00127-023-02481-5 (PMC10960744; doi:10.1007/s00127-023-02481-5)
Supplement: Supplementary file 2 — Supplementary file2 (PDF 115 KB) [file 127_2023_2481_MOESM2_ESM.pdf]

Online Resource 2. Calculating Gini-Simpson Diversity Index (GSDI).

Author information: Mental health service diversity and work disability: associations of mental health service system characteristics and mood disorder disability pensioning in Finland. (2023). Social Psychiatry and Psychiatric Epidemiology. Tino Karolaakso [Faculty of Social Sciences (Psychology), Tampere University, Arvo Ylpön katu, 34, 33520 Tampere, Finland; tino.karolaakso@tuni.fi], Reija Autio, Petra Suontausta. Helena Leppänen, Päivi Rissanen, Turkka Näppilä, Martti T. Tuomisto, Sami Pirkola.

The Simpson's index *D* (Simpson, 1949) means the probability that any two individuals randomly picked from an infinitely large population will belong to the same species or class. Commonly it is applied as (1 – *D*), which is known as the Gini-Simpson Diversity Index (GSDI). The GSDI thus measures the probability that two randomly picked individuals belong to different species. GSDI is also known as the Gibbs-Martin index or the Blau index in psychology sociology, and management studies.

The GSDI values were calculated using R version 4.0.1 (R Core Team, 2020) and the R-package *diverse* (Guevara, M. R., Hartmann, D., Mendoza, 2016).

For a given area, and in this study for one municipality, the GSDI was calculated as follows:

$$1 - \sum_i (p_i^2)$$

where:

- p_i is the "species" (ESMS-R class) proportion, $p_i = x_i \; / \; N$.
- N is the total number of individuals (in this case the available MHS units) in the community (municipality)

By combining and weighing service richness with the number of available units in the ESMS-R class, the GSDI defines an index of 0 to 1 for the municipalities' MHS diversity. A GSDI close to 1 signifies that there are several ESMS-R classes in the municipality, and the available MHS unit proportion of ESMS-R classes is even. A low GSDI indicates that the municipality's MHS is not diverse. For instance, if there is only one class of MHS according to the ESMS-R taxonomy in the municipality, the GSDI is 0.

Example: Municipality X has 44 MHS units available to its population. These 44 MHS units belong to 18 different ESMS-R classes, so the service richness of X is 18. Of these services, D1.2 has one MHS unit, D3.2 has three MHS units, O10.1. has seven MHS units, R2 has six MHS units, and so on.

The formula for calculating the GSDI for X is:

$$1 - \left((1/44)^2 + (3/44)^2 + (7/44)^2 + (6/44)^2 + (1/44)^2 + (3/44)^2 + (1/44)^2 + (2/44)^2 + (2/44)^2 + (2/44)^2 + (1/44)^2 + (1/44)^2 + (1/44)^2 + (1/44)^2 + (7/44)^2 + (1/44)^2 + (2/44)^2 + (2/44)^2\right)$$

= GSDI is approximately 0.9070248
