# Supplementary material for: Mental health service diversity and work disability: associations of mental health service system characteristics and mood disorder disability pensioning in Finland
Source: Soc Psychiatry Psychiatr Epidemiol. 2023 Apr 28;59(4):631–42. doi: 10.1007/s00127-023-02481-5 (PMC10960744; doi:10.1007/s00127-023-02481-5)
Supplement: Supplementary file 3 — Supplementary file3 (PDF 632 KB) [file 127_2023_2481_MOESM3_ESM.pdf]

Online Resource 3. Clustered heatmaps of the correlation between MHS factors as well as sociodemographic and -economic factors in all municipalities.

Author information: Mental health service diversity and work disability: associations of mental health service system characteristics and mood disorder disability pensioning in Finland. (2023). Social Psychiatry and Psychiatric Epidemiology. Tino Karolaakso [Faculty of Social Sciences (Psychology), Tampere University, Arvo Ylpön katu, 34, 33520 Tampere, Finland; tino.karolaakso@tuni.fi], Reija Autio, Petra Suontausta. Helena Leppänen, Päivi Rissanen, Turkka Näppilä, Martti T. Tuomisto, Sami Pirkola.

All municipalities

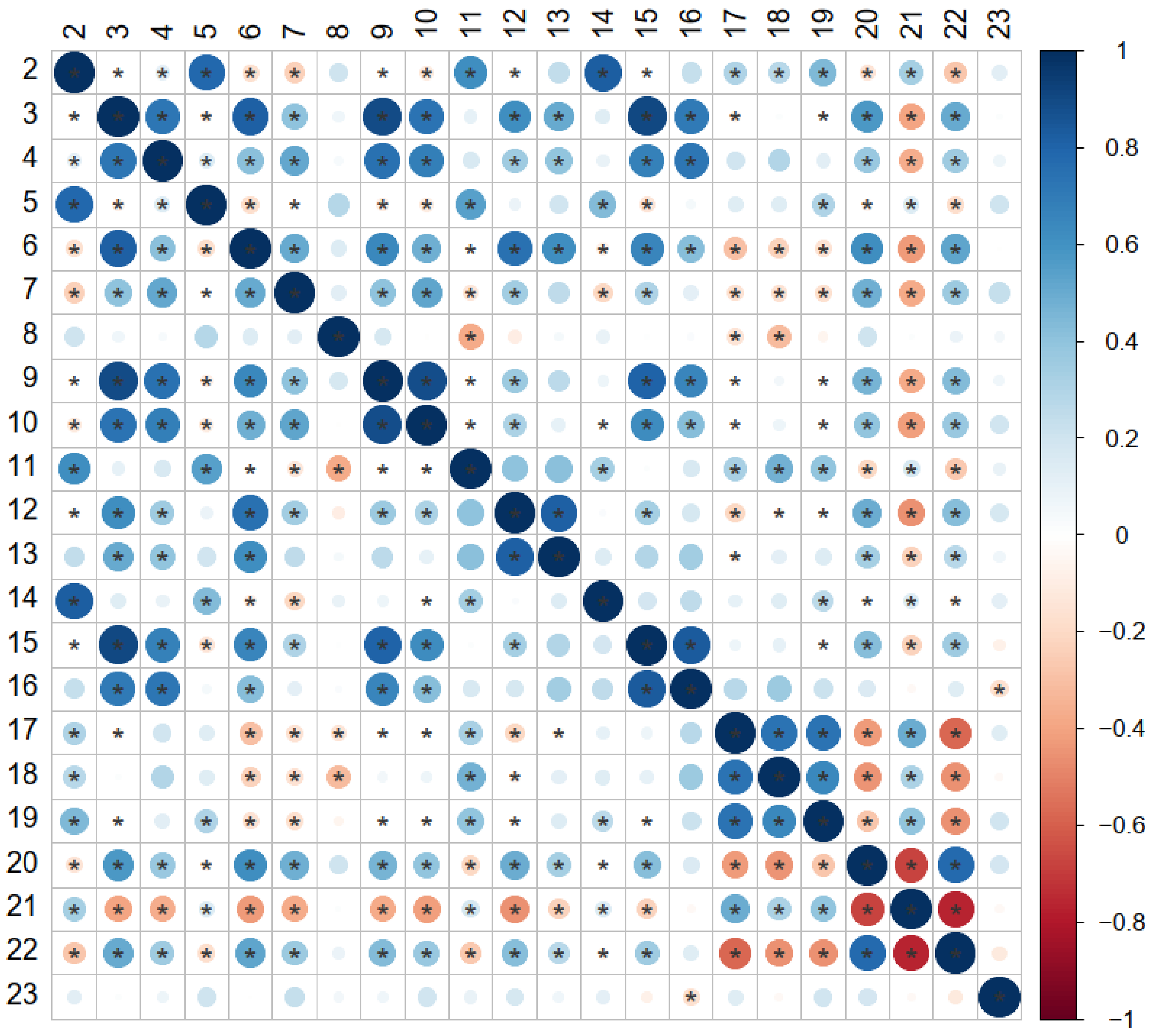

Urban municipalities

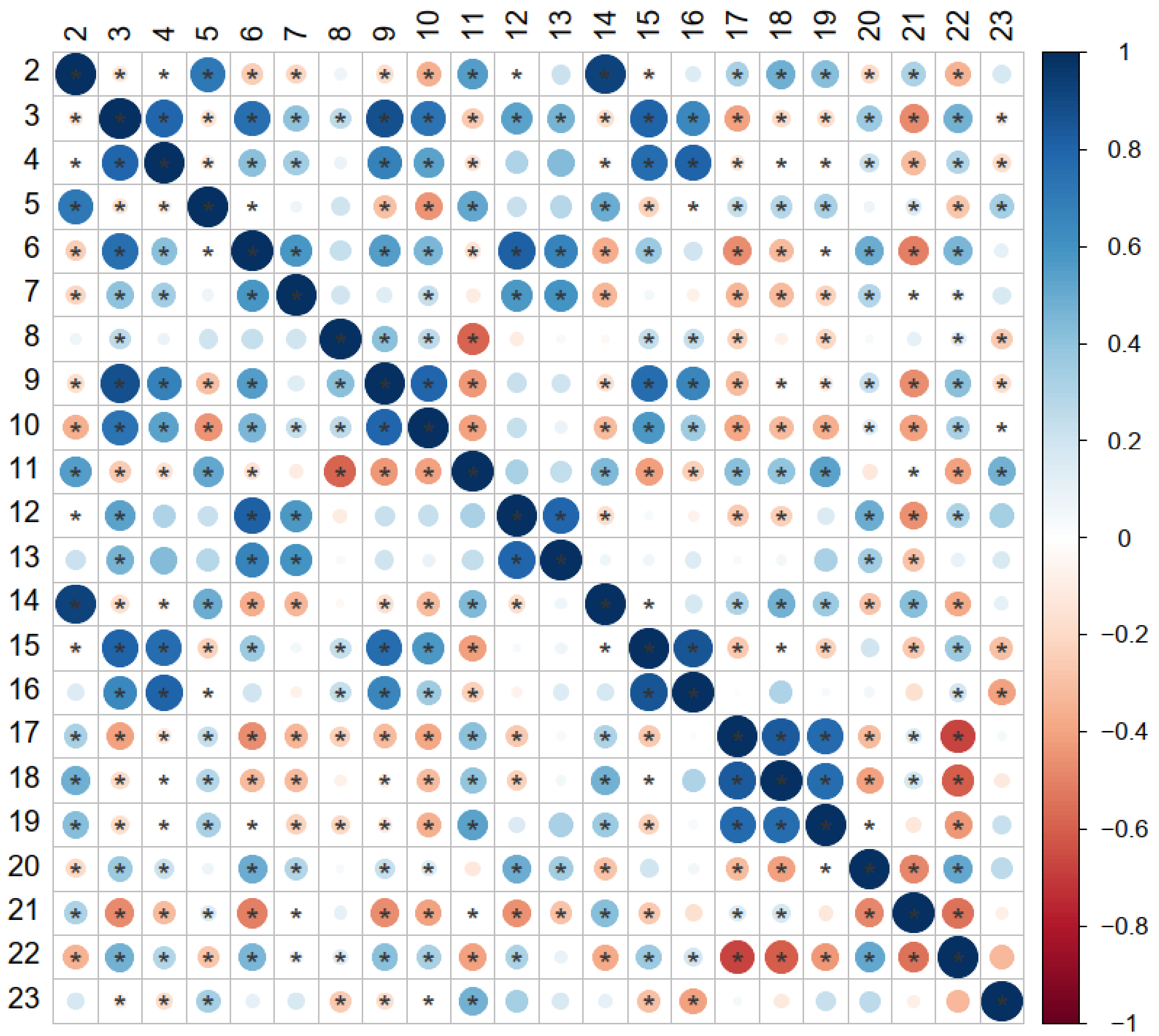

Semi-urban municipalities

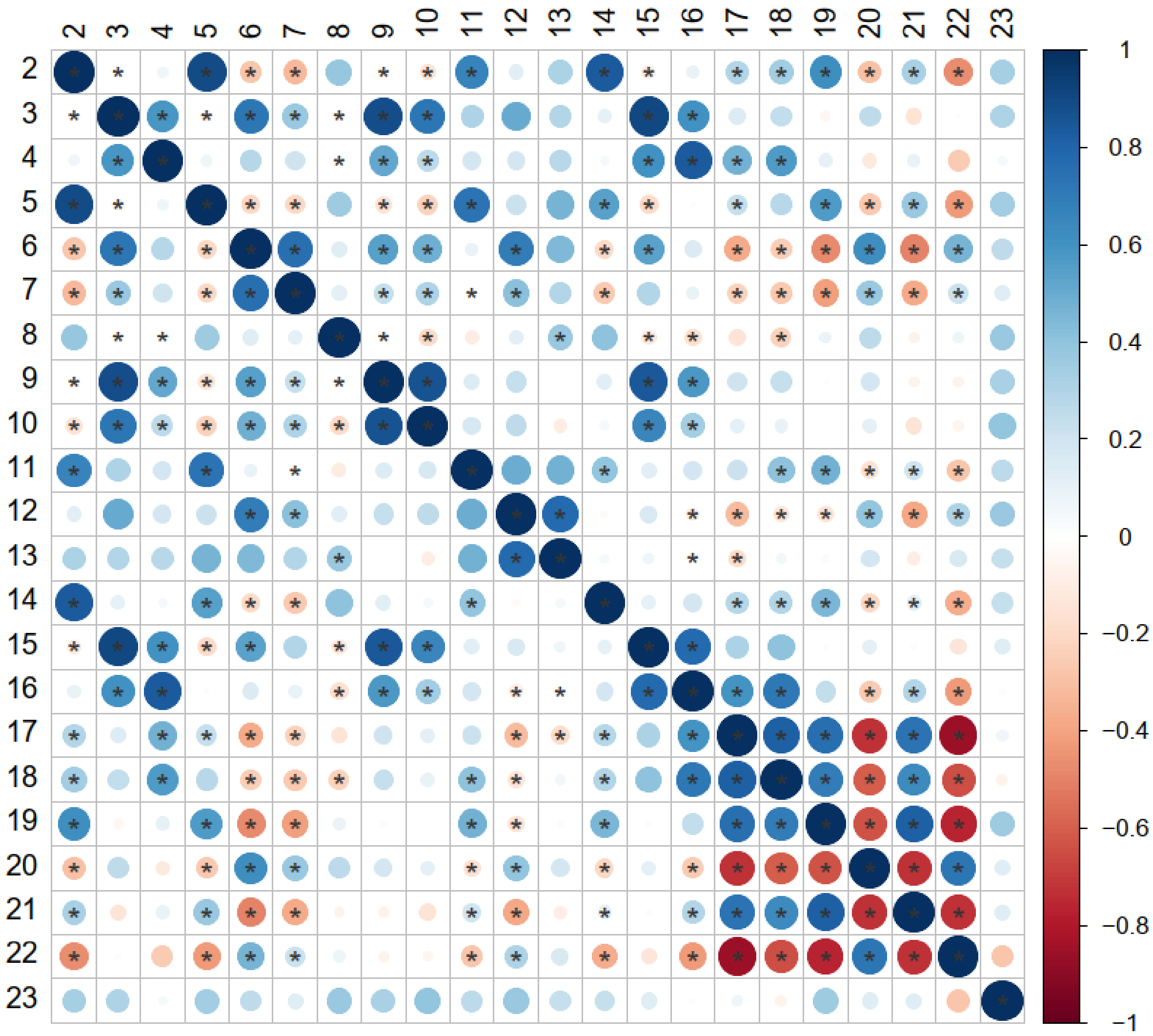

Rural municipalities

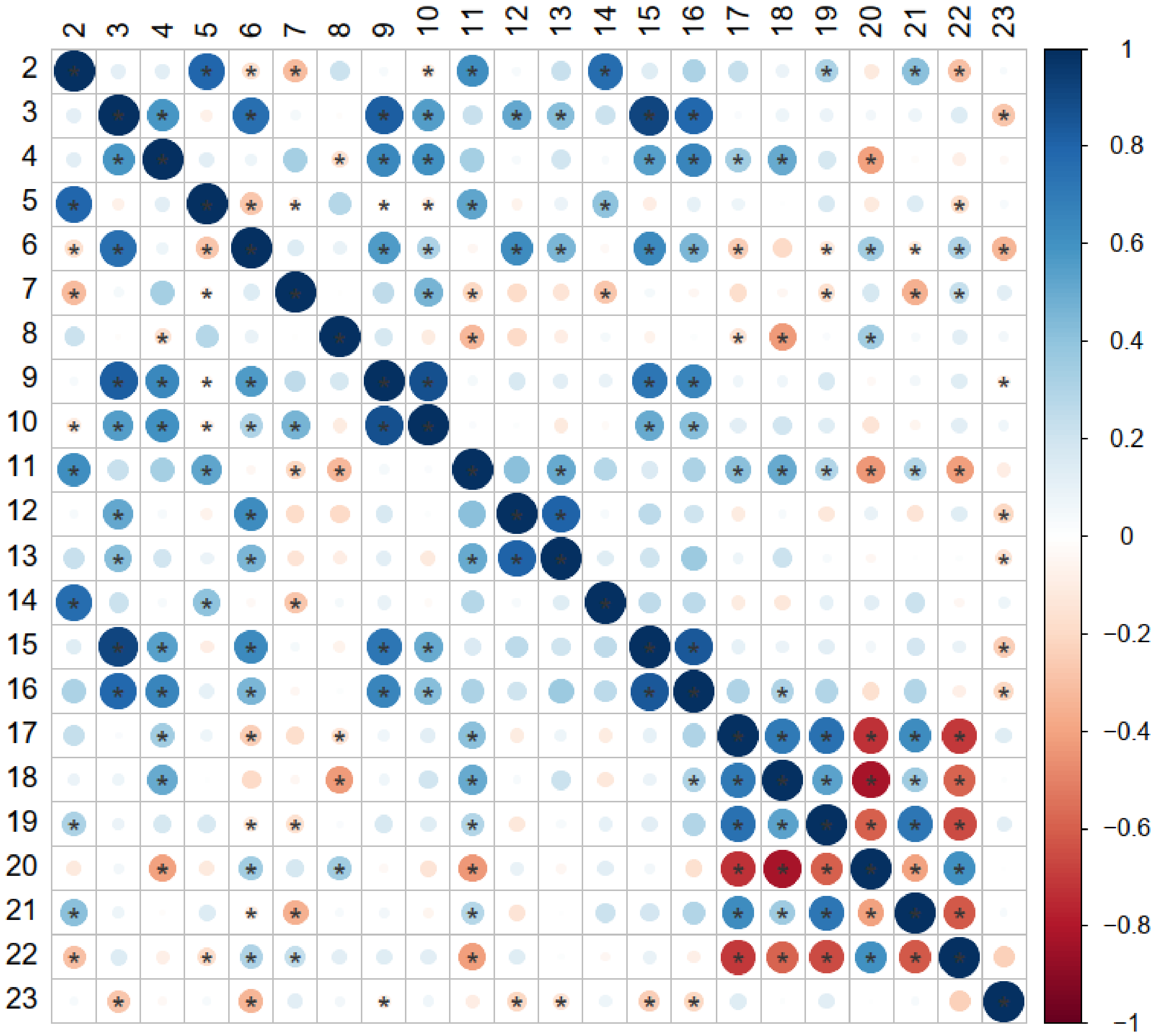

2. MHS FTE resources per 1000 inhabitants
3. MHS Service richness
4. MHS Service diversity
5. O FTE resources per 1000 inhabitants
6. O Service richness
7. O Service diversity
8. Without gatek. FTE resources per 1000 inhabitants
9. Without gatek. Service richness

10. Without gatek. Service diversity
11. With gatek. FTE resources per 1000 inhabitants
12. With gatek. Service richness
13. With gatek. Service diversity
14. Centralized FTE resources per 1000 inhabitants
15. Centralized Service richness
16. Centralized Service diversity
17. Mental health index, not age-standardized
18. Unemployment rate, as % of total population
19. Household-dwelling-units with one person, as % of all household/dwelling-units
20. Population density, population/km2
21. Demographic dependency ratio, as the number of people aged under 15 and over 64 per hundred working-age people aged 15-64
22. Higher education qualifications, as % of total population aged 20 and over
23. Not in education or training aged 17-24, as % of total population of same age
