# Supplementary material for: Mental health service diversity and work disability: associations of mental health service system characteristics and mood disorder disability pensioning in Finland
Source: Soc Psychiatry Psychiatr Epidemiol. 2023 Apr 28;59(4):631–42. doi: 10.1007/s00127-023-02481-5 (PMC10960744; doi:10.1007/s00127-023-02481-5)
Supplement: Supplementary file 4 — Supplementary file4 (PDF 241 KB) [file 127_2023_2481_MOESM4_ESM.pdf]

Online Resource 4. Characteristics of the municipality-level mental health service ESMS-R factors in Finland as boxplot figures.

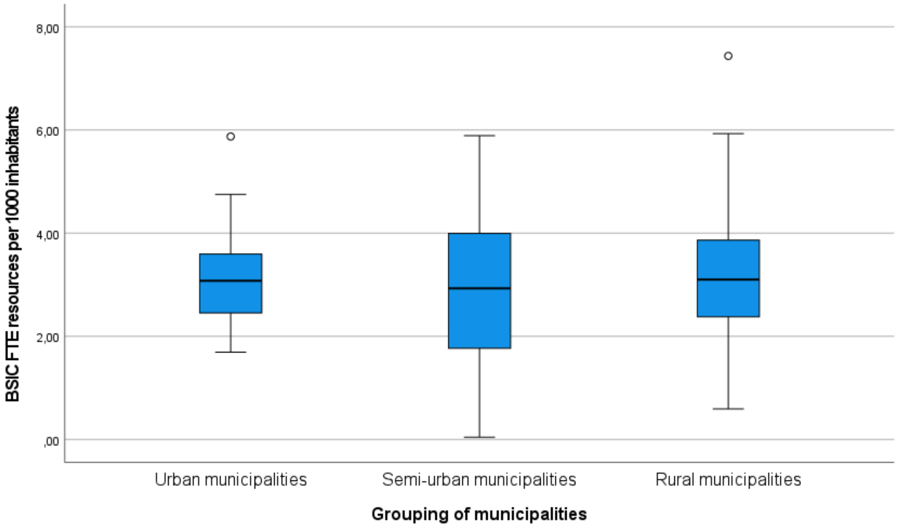

Author information: Mental health service diversity and work disability: associations of mental health service system characteristics and mood disorder disability pensioning in Finland. (2023). Social Psychiatry and Psychiatric Epidemiology. Tino Karolaakso [Faculty of Social Sciences (Psychology), Tampere University, Arvo Ylpön katu, 34, 33520 Tampere, Finland; tino.karolaakso@tuni.fi], Reija Autio, Petra Suontausta. Helena Leppänen, Päivi Rissanen, Turkka Näppilä, Martti T. Tuomisto, Sami Pirkola.

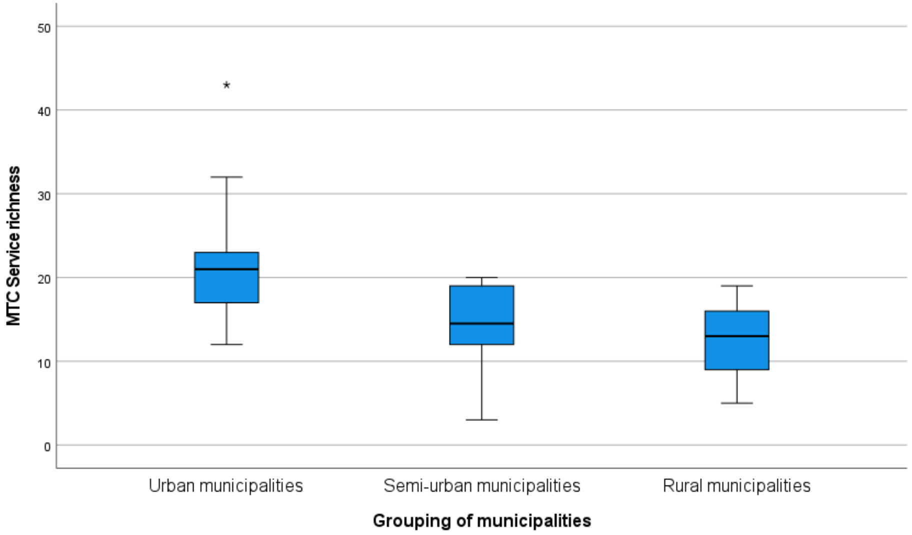

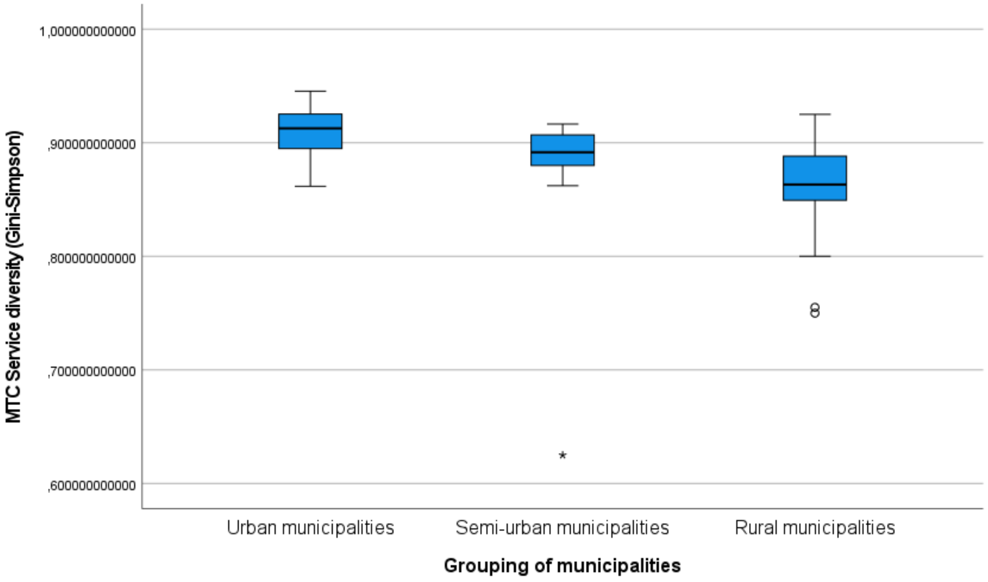

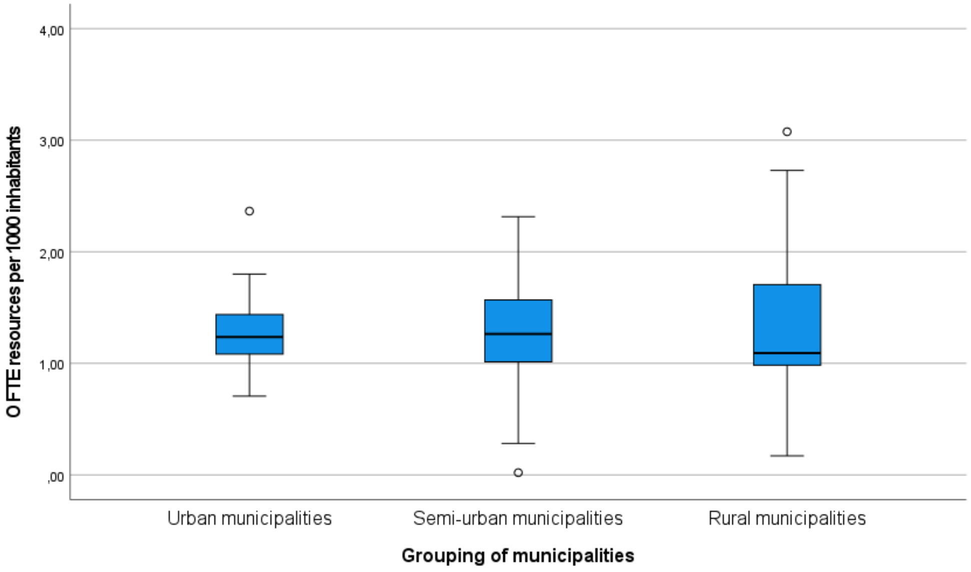

O FTE resources per 1000 inhabitants
4,00
3,00
2,00
1,00
,00
Urban municipalities
Semi-urban municipalities
Rural municipalities
Grouping of municipalities

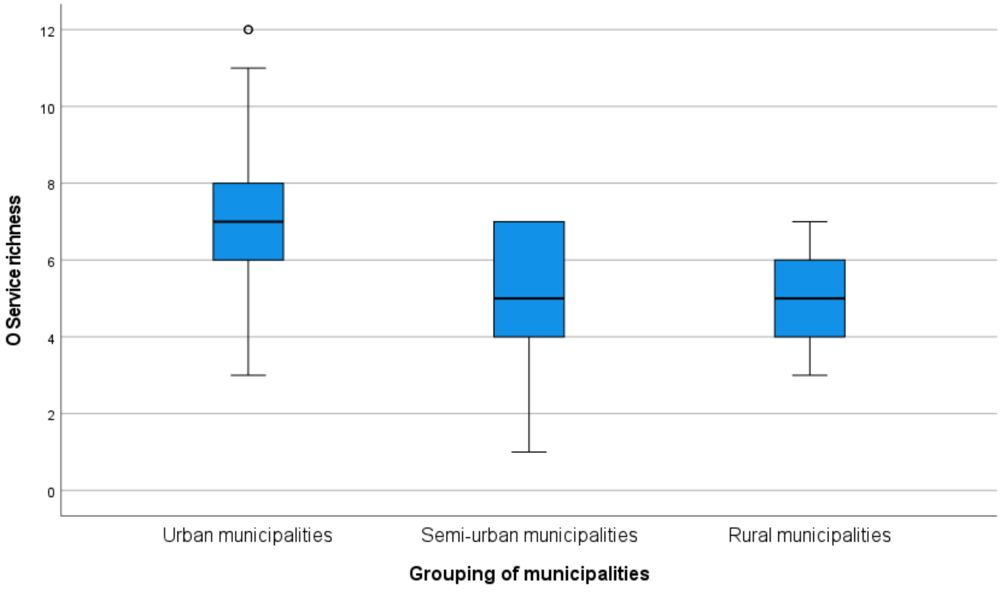

O Service richness
12
10
8
6
4
2
0
Urban municipalities
Semi-urban municipalities
Rural municipalities
Grouping of municipalities

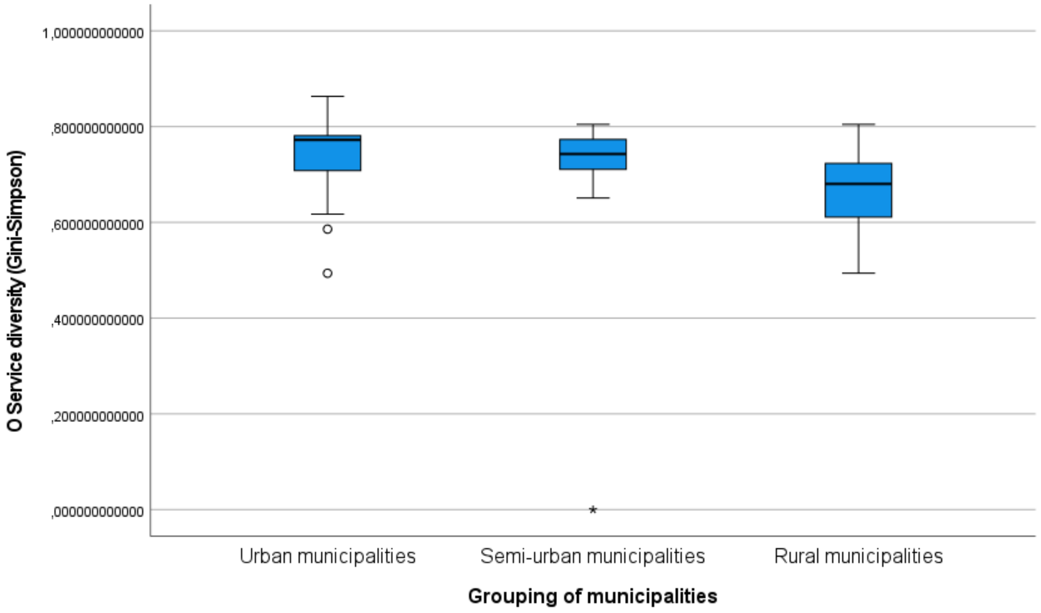

O Service diversity (Gini-Simpson)
1,000000000000
,800000000000
,600000000000
,400000000000
,200000000000
,000000000000
Urban municipalities
Semi-urban municipalities
Rural municipalities
Grouping of municipalities

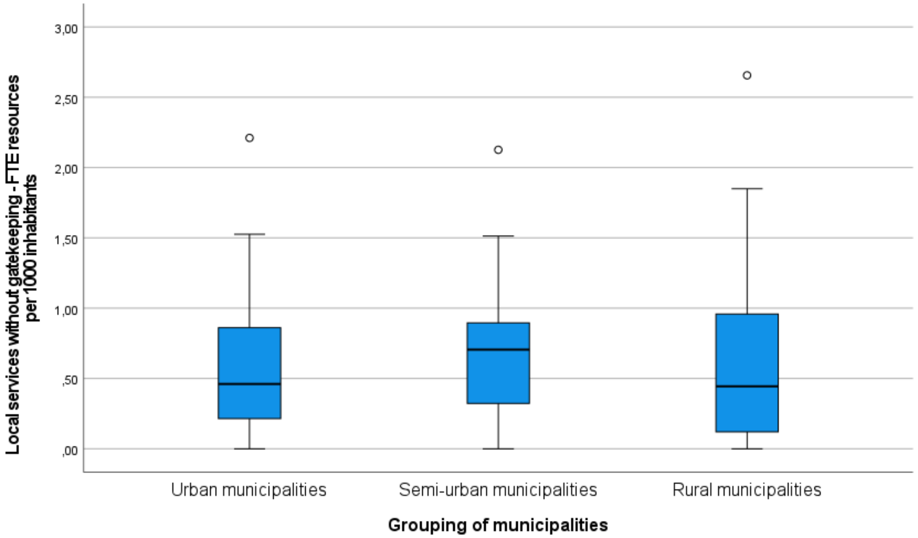
Local services without gatekeeping - FTE resources per 1000 inhabitants
3,00
2,50
2,00
1,50
1,00
,50
,00
Urban municipalities
Semi-urban municipalities
Rural municipalities
Grouping of municipalities

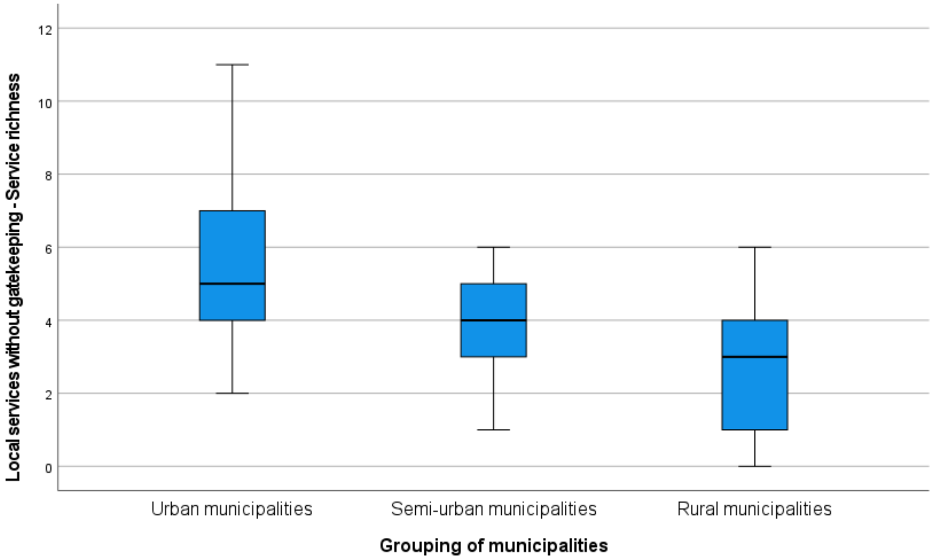
Local services without gatekeeping - Service richness
12
10
8
6
4
2
0
Urban municipalities
Semi-urban municipalities
Rural municipalities
Grouping of municipalities

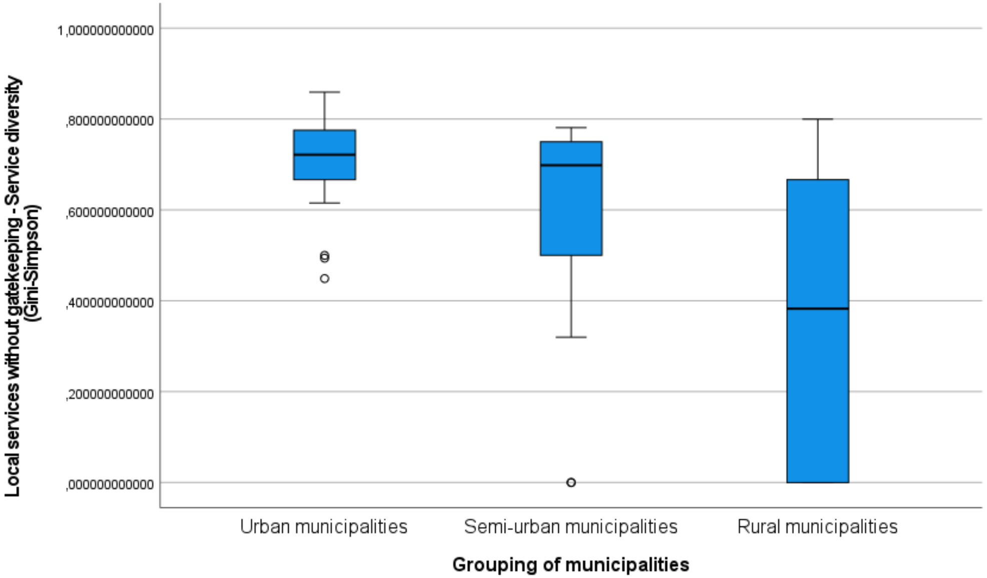
Local services without gatekeeping - Service diversity (Gini-Simpson)
1,000000000000
,800000000000
,600000000000
,400000000000
,200000000000
,000000000000
Urban municipalities
Semi-urban municipalities
Rural municipalities
Grouping of municipalities

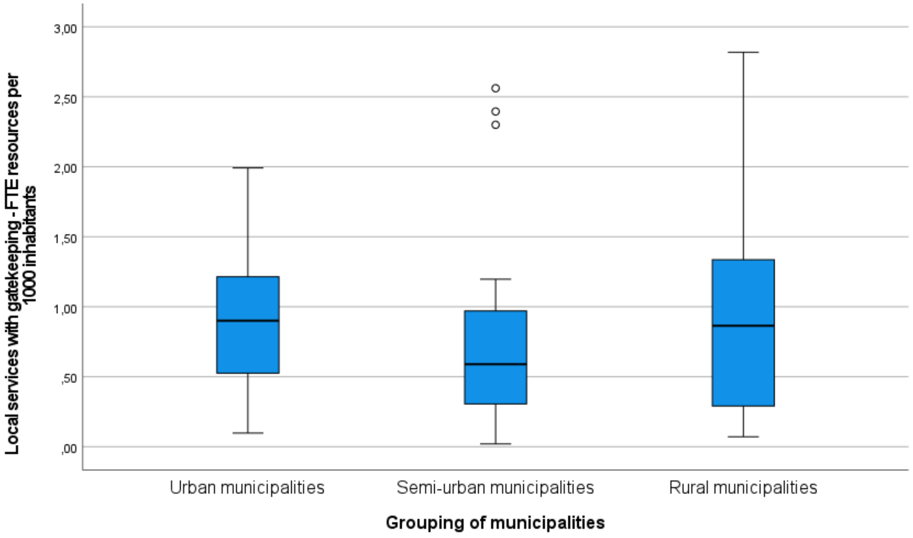
Local services with gatekeeping - FTE resources per 1000 inhabitants
3,00
2,50
2,00
1,50
1,00
,50
,00
Urban municipalities
Semi-urban municipalities
Rural municipalities
Grouping of municipalities

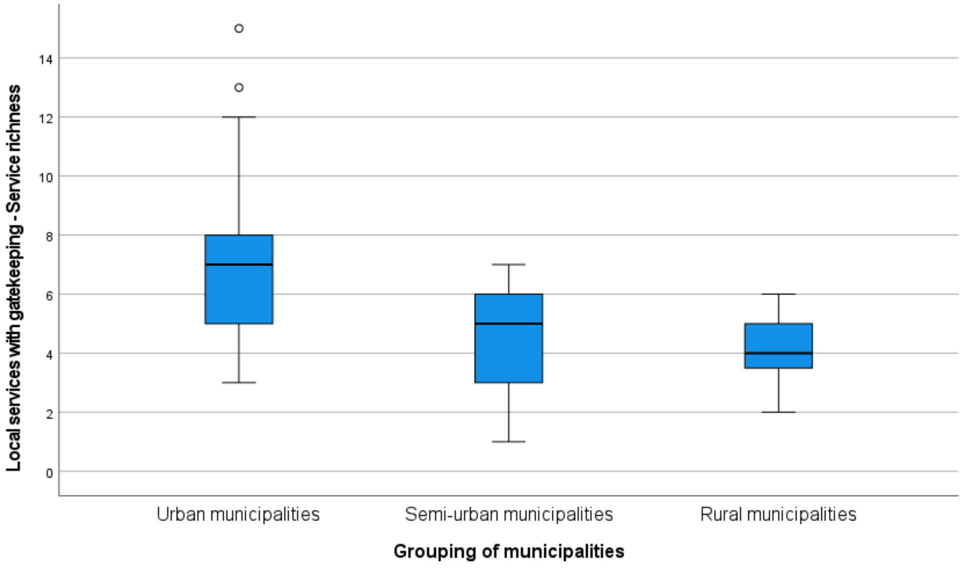
Local services with gatekeeping - Service richness
14
12
10
8
6
4
2
0
Urban municipalities
Semi-urban municipalities
Rural municipalities
Grouping of municipalities

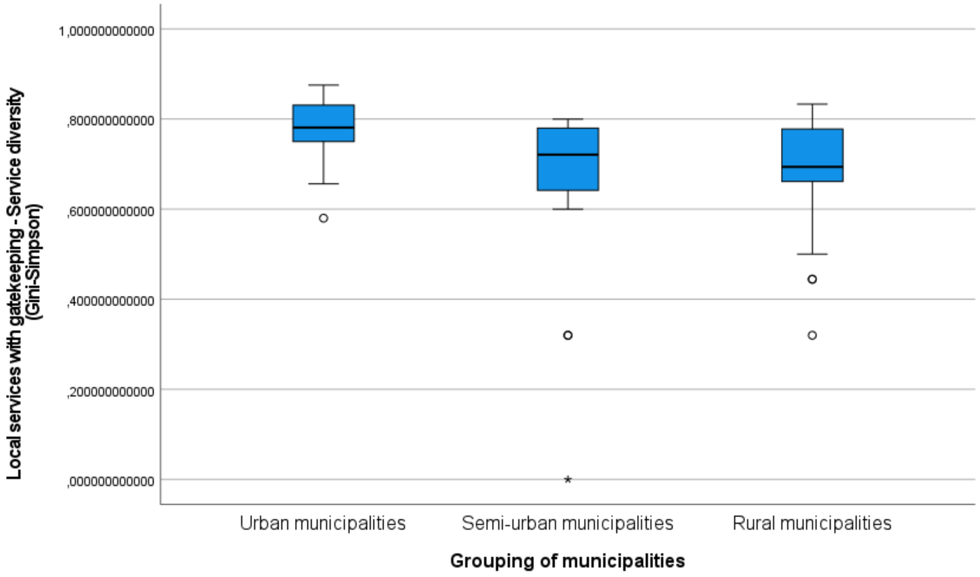
Local services with gatekeeping - Service diversity (Gini-Simpson)
1,000000000000
,800000000000
,600000000000
,400000000000
,200000000000
,000000000000
Urban municipalities
Semi-urban municipalities
Rural municipalities
Grouping of municipalities

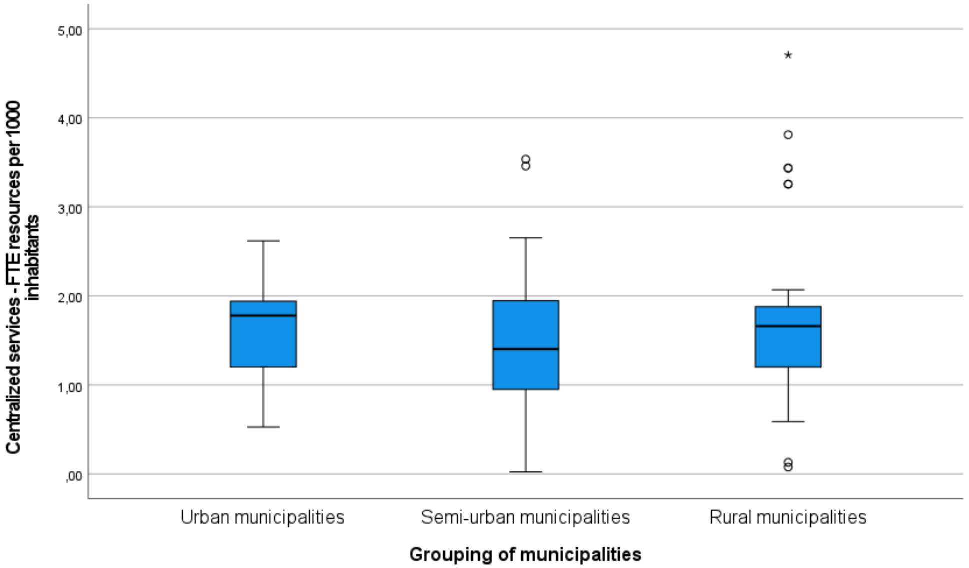
Centralized services - FTE resources per 1000 inhabitants
5,00
4,00
3,00
2,00
1,00
,00
Urban municipalities
Semi-urban municipalities
Rural municipalities
Grouping of municipalities

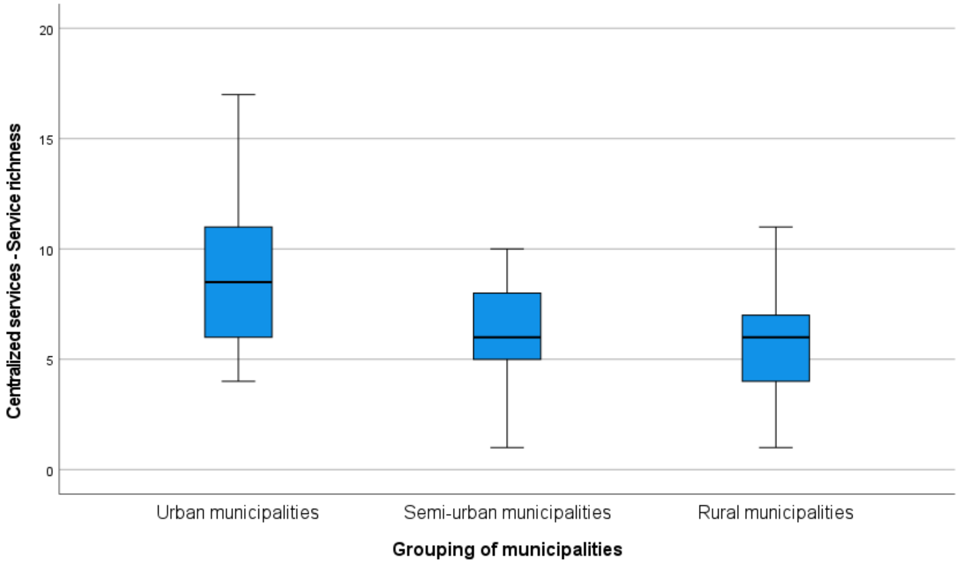
Centralized services - Service richness
20
15
10
5
0
Urban municipalities
Semi-urban municipalities
Rural municipalities
Grouping of municipalities

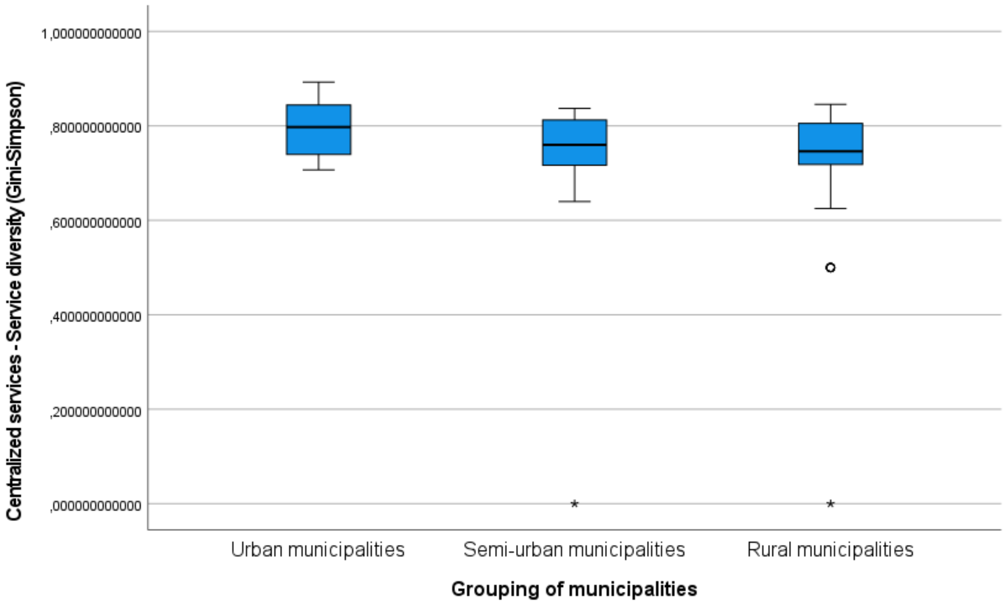
Centralized services - Service diversity (Gini-Simpson)
1,000000000000
,800000000000
,600000000000
,400000000000
,200000000000
,000000000000
Urban municipalities
Semi-urban municipalities
Rural municipalities
Grouping of municipalities
